# Placental ^13^C-DHA metabolism and relationship with maternal BMI, glycemia and birthweight

**DOI:** 10.1186/s10020-021-00344-w

**Published:** 2021-08-06

**Authors:** Oliver C. Watkins, Preben Selvam, Reshma Appukuttan Pillai, Victoria K. B. Cracknell-Hazra, Hannah E. J. Yong, Neha Sharma, Amaury Cazenave-Gassiot, Anne K. Bendt, Keith M. Godfrey, Rohan M. Lewis, Markus R. Wenk, Shiao-Yng Chan

**Affiliations:** 1 Department of Obstetrics and Gynaecology, Yong Loo Lin School of Medicine, National University of Singapore, National University Health System, 1E Kent Ridge Road, NUHS Tower Block, Level 12, Singapore, 119228 Singapore; 2 Singapore Institute for Clinical Sciences, Agency for Science, Technology and Research, Singapore, Singapore; 3 Department of Biochemistry, Yong Loo Lin School of Medicine, National University of Singapore, Singapore, Singapore; 4 Singapore Lipidomics Incubator, Life Sciences Institute, National University of Singapore, Singapore, Singapore; 5 MRC Lifecourse Epidemiology Unit and NIHR Southampton Biomedical Research Centre, University of Southampton and University Hospital Southampton NHS Foundation Trust, Southampton, UK

**Keywords:** Pregnancy, Placenta, Lipid, Stable-isotope, Gestational diabetes, LCMS

## Abstract

**Background:**

Fetal docosahexaenoic acid (DHA) supply relies on preferential transplacental transfer, which is regulated by placental DHA lipid metabolism. Maternal hyperglycemia and obesity associate with higher birthweight and fetal DHA insufficiency but the role of placental DHA metabolism is unclear.

**Methods:**

Explants from 17 term placenta were incubated with ^13^C-labeled DHA for 48 h, at 5 or 10 mmol/L glucose treatment, and the production of 17 individual newly synthesized ^13^C-DHA labeled lipids quantified by liquid chromatography mass spectrometry.

**Results:**

Maternal BMI positively associated with ^13^C-DHA-labeled diacylglycerols, triacylglycerols, lysophospholipids, phosphatidylcholine and phosphatidylethanolamine plasmalogens, while maternal fasting glycemia positively associated with five ^13^C-DHA triacylglycerols. In turn, ^13^C-DHA-labeled phospholipids and triacylglycerols positively associated with birthweight centile. In-vitro glucose treatment increased most ^13^C-DHA-lipids, but decreased ^13^C-DHA phosphatidylethanolamine plasmalogens. However, with increasing maternal BMI, the magnitude of the glucose treatment induced increase in ^13^C-DHA phosphatidylcholine and ^13^C-DHA lysophospholipids was curtailed, with further decline in ^13^C-DHA phosphatidylethanolamine plasmalogens. Conversely, with increasing birthweight centile glucose treatment induced increases in ^13^C-DHA triacylglycerols were exaggerated, while glucose treatment induced decreases in ^13^C-DHA phosphatidylethanolamine plasmalogens were diminished.

**Conclusions:**

Maternal BMI and glycemia increased the production of different placental DHA lipids implying impact on different metabolic pathways. Glucose-induced elevation in placental DHA metabolism is moderated with higher maternal BMI. In turn, findings of associations between many DHA lipids with birthweight suggest that BMI and glycemia promote fetal growth partly through changes in placental DHA metabolism.

**Supplementary Information:**

The online version contains supplementary material available at 10.1186/s10020-021-00344-w.

## Background

The placenta regulates fetal nutritional supply and communication across the maternal–placental–fetal axis (Haggarty 2010; Lewis et al. 2018). DHA-containing lipids are important for fetal growth and development (Knopp et al. 1998; Higa and Jawerbaum 2013; Khan 2007), and the fetus relies on placental transfer of maternal DHA since the fetal–placental unit has limited synthetic capability (Hanebutt et al. 2008). Transplacental DHA supply is regulated by placental lipid metabolism, but there is limited understanding of the specific pathways involved (Haggarty 2010; Lewis et al. 2018). Understanding these mechanisms could pave the way to optimizing in-utero DHA supply for fetal development and designing strategies to rectify possible fetal DHA insufficiency in pathological conditions such as gestational diabetes (GDM) and maternal obesity (Haggarty 2010; Harris and Baack 2015).

Excessive fetal growth and adiposity, which are more prevalent with GDM and maternal obesity, increases risk of birth trauma, neonatal morbidity and perinatal mortality, as well as long term cardiometabolic disease (Leng et al. 2015; Boney et al. 2005; Catalano 2003). Lipids serve as metabolic fuel for the fetus and variations in fat accretion contribute to birthweight differences (Catalano 2003). However, fetal development also depends on less abundant, but highly bioactive DHA-lipids, which impact processes such as placental nutrient uptake, storage and export (Larqué et al. 2014; Herrera and Ortega-Senovilla 2018a; Delhaes et al. 2018; Duttaroy 2016). DHA-lipids modulate lipid membrane properties, transport, and membrane-related-processes such as G-Protein-coupled and interleukin-2 receptor-signaling (Li et al. 2005; Mitchell et al. 2003) and lipid droplet formation (Lecchi et al. 2013; Digel et al. 2010; Onuki et al. 2006); processes which regulate placental transfer of nutrients (Kolahi et al. 2016; Saito 2001). DHA and related derivatives also act as signaling molecules (Larqué et al. 2014; Herrera and Ortega-Senovilla 2018a; Delhaes et al. 2018; Gallo et al. 2017) that regulate fetal growth, inflammatory processes and labor onset (Duttaroy 2016; Rogers et al. 2013; Farhat et al. 2020), and protect against oxidative stress (Yavin et al. 2002; Lessig and Fuchs 2009; Wang and Wang 2010).

DHA is particularly important for fetal brain growth and neurological development (Helland et al. 2008; Steenweg-de Graaff et al. 2015; Wainwright 2002) while circulating maternal DHA in late pregnancy is also associated with fetal insulin sensitivity and long-term future weight gain for the child (Zhao et al. 2014; Moon et al. 2013). However, factors contributing to differences in levels of fetal–placental DHA lipids and mechanisms by which they may affect growth and development are poorly understood.

Alterations in placental and cord DHA lipids (Meher et al. 2016; Foreman-van Drongelen et al. 1995; Felton et al. 1994; Hellmuth et al. 2017; Patel et al. 2017) have indeed been linked with birthweight, likely predominantly due to the signaling actions of DHA-lipids. The directionality of associations is lipid-dependent, with cord phospholipid and lyso-phospholipid DHA positively linked with birthweight (Foreman-van Drongelen et al. 1995; Felton et al. 1994; Patel et al. 2017), and un-esterified cord DHA negatively linked (Hellmuth et al. 2017). This is consistent with the idea that different DHA-derivatives, may signal different effects.

The placenta selectively and preferentially transfers DHA leading to higher DHA content in umbilical cord blood compared to the maternal circulation (Larqué et al. 2014; Haggarty 2002; Pagán et al. 2013). Transplacental DHA transfer is substantially regulated by placental lipid metabolism, with compartmentalisation between different lipid pools influencing the bio-availability of un-esterified DHA and related compounds for placental processes and fetal supply (Lewis et al. 2018; Kolahi et al. 2016,2018). Although not well understood, accessibility likely depends on both lipid location and lipid type. For example, DHA stored in placental lipid droplets is more accessible than DHA in lipid-membrane structures important for cell function (Kolahi et al. 2016,2015). Meanwhile, triacylglycerols (TGs) undergo turnover more quickly than phospholipids suggesting that TG metabolism could be an important regulator of the availability of free un-esterified fatty acids (Watkins et al. 2019a). Furthermore, placental DHA-phosphatidyl-ethanolamine (DHA-PE), rather than DHA-phosphatidylcholine (DHA-PC) may be a more important source of DHA for the fetus (Uhl et al. 2015). The actual DHA-lipid forms that are being transferred from the placenta to the fetus (as signaling agents or building blocks) may include non-esterified DHA, other forms such as lysophospholipids which can cross transmembrane channels like MFSD2A, or by exocytosis of vesicles. Hence, tracking the metabolism of individual DHA-containing lipid species is necessary for understanding transplacental DHA supply and placental DHA-lipid action.

Both pre-pregnancy BMI and GDM have been negatively associated with DHA-phospholipids in umbilical cord blood erythrocytes (Thomas et al. 2005; Wijendran et al. 2000), whilst GDM has also been associated with reduced DHA in cord plasma (Zornoza-Moreno et al. 2014; Ortega-Senovilla et al. 2009), but other studies reported no difference (Thomas et al. 2005; Wijendran et al. 2000). Studies on endogenous ^12^C-DHA, and in-vivo studies using ^13^C-DHA, have shown that transplacental DHA transfer and consequent enrichment of cord plasma with DHA-lipids is impaired in GDM pregnancies and with obesity, suggesting placental dysfunction as a possible contributor to fetal DHA deficiency (Pagán et al. 2013; Wijendran et al. 2000; Visiedo et al. 2015; Gázquez et al. 2020). While functional studies in isolated primary human trophoblasts demonstrated impaired placental DHA uptake with GDM (Araújo et al. 2013), descriptive studies of GDM placental biopsies found elevated DHA-lipids (Uhl et al. 2015; Bitsanis et al. 2006). Since isolated primary human trophoblasts exposed to GDM-like conditions in-vitro showed decreased DHA transfer across a trophoblast monolayer, increased TG content, and decreased expression of proteins which suppress lipid synthesis, it was hypothesized that reduced DHA transfer to the fetus in GDM could result from increased placental DHA-lipid synthesis and retention as well as impaired uptake (Mishra et al. 2020). Indeed, DHA-phospholipids were previously found to be elevated in placenta in GDM or obesity (Uhl et al. 2015; Bitsanis et al. 2006) but reduced in the fetal compartment (Thomas et al. 2005; Wijendran et al. 2000) suggestive of placental DHA retention, while placental TGs in general are increased with hyperglycemia (Visiedo et al. 2015; Visiedo et al. 2013; Mishra et al. 2020; Tewari et al. 2011). However, these studies have not directly measured DHA-lipid synthesis, nor fully characterized the specific DHA-containing lipids involved.

Our present in-vitro study measures the fresh production of specific ^13^C-DHA-labeled lipids by placental explants from ^13^C-labeled-DHA. ^13^C-DHA is not naturally present in placenta, but is metabolized identically to naturally occurring ^12^C-DHA. Measured ^13^C-DHA-lipids therefore reflect the capacity of the placenta to take up, synthesize and metabolize new individual DHA-lipids, details which were not possible to assess with older methods involving radiolabeled ^14^C-DHA that could, at best, only measure overall uptake into broad extractable fractions such as total phospholipids by quantifying beta radiation (Araújo et al. 2013). Furthermore, the effects of in-vivo confounding, such as maternal dietary intake, maternal and fetal tissue DHA metabolism, circulating lipid concentrations, and placental blood flow, are all reduced in an in-vitro explant model, thus findings of ^13^C-DHA lipids are reflective of placental tissue DHA uptake and metabolism in isolation.

Current evidence therefore suggests that placental lipid metabolism is important for preferential transplacental DHA transfer, and that in GDM or maternal obesity some DHA lipids may be reduced in the fetal compartment (Uhl et al. 2015; Bitsanis et al. 2006) but elevated in placenta, suggestive of placental DHA retention. Given the reported lipid-dependent links between placental and cord blood DHA lipids with birthweight (Helland et al. 2008; Steenweg-de Graaff et al. 2015; Wainwright 2002; Meher et al. 2016; Varastehpour et al. 2006), characterizing the specific alterations in individual placental DHA lipids could uncover specific lipidomic mechanisms by which increasing maternal glycemia and BMI may lead to dysregulated fetal growth commonly observed with these conditions.

Thus, we hypothesized that placental DHA metabolism is programed by maternal BMI and antenatal glycemia, leading to alterations in placental DHA-lipids, which could in turn promote fetal growth and birthweight. We aimed to establish the association between maternal BMI and glycemia with the production and metabolism of individual placental ^13^C-DHA-lipids. We determined the direct effects of glucose treatment in-vitro on placental ^13^C-DHA lipid metabolism, and how this response to glucose is modulated by prior programing from in-vivo exposure to higher maternal BMI and glycemia. Relationships between placental ^13^C-DHA-lipids and birthweight were then examined alongside interactions with maternal BMI and glycemia.

## Methods

### Recruitment and clinical characteristics of participants

Placenta (n = 17) were collected from women recruited at the National University Hospital, Singapore with informed written consent. Universal screening for gestational diabetes (GDM) was performed at mid-gestation by a three time-point 75 g oral glucose tolerance test (OGTT) using WHO 2013 criteria. Cases of known pre-existing type 1 and type 2 diabetes mellitus were excluded as well as cases of possible pre-existing diabetes defined by antenatal OGTT results of fasting glycemia ≥ 7 mmol/L or 2 h glycemia ≥ 11.1 mmol/L. Non-smoking mothers who delivered after 37 weeks’ gestation by elective Caesarean section, with newborns who were greater than the 10th percentile (by local sex- and gestational age-standardized references (Mikolajczyk et al. 2011; Ong et al. 2020); this would exclude cases of potential malplacentation and uteroplacental insufficiency) were eligible. Women with GDM (n = 9) and normoglycemia (non-GDM; n = 8) were recruited and matched for first trimester BMI (similar mean (SD) kg/m^2^: GDM: 24.1 (3.9); non-GDM: 26.6 (5.0)), hence neither fasting glycemia nor post-load glycemia (by OGTT) were significantly associated with BMI in this study population. This selection strategy ensured a balance of cases across the more physiological glycemic range and across a range of maternal BMIs for these mechanistic studies of placental DHA lipid metabolism. Researchers were blinded to the clinical characteristics of the women and neonates involved until the completion of all explant culture experiments and LCMS. Table 1 shows the characteristics of the study population (individual details in Additional file 1) as extracted from medical records.

**Table 1:** Clinical characteristics of study population

Clinical characteristics	Total (n = 17)	Non-GDM (n = 8)	GDM (n = 9)
Maternal age (years)	33.4 (2.9)	32.5 (2.6)	34.1 (3.2)
Chinese: Indian ethnicity	11 (65%): 6 (35%)	6 (75%): 2 (25%)	5 (56%): 4 (44%)
Maternal BMI in first trimester (kg/m^2^)	25.4 (4.6)	24.1 (3.9)	26.6 (5)
Normal weight: Overweight: Obese^a^	5 (29%): 6 (35%): 6 (35%)	3 (38%): 2 (25%): 3 (38%)	2 (22%): 4 (44%): 3 (33%)
Fasting glycemia (mmol/L)^b^	4.5 (0.5)	4.4 (0.2)	4.6 (0.6)
1-h glycemia (mmol/L)^b^	9.0 (1.7)	7.8 (1.4)	10.1 (1.15)
2-h glycemia (mmol/L)^b^	7.6 (1.6)	6.4 (0.9)	8.8 (1.2)
Antenatal PUFA-containing supplementation	6 (35%)	3 (33%)	3 (38%)
Gestational age at delivery (days)	271.4 (5.9)	273.3 (7.4)	269.7 (3.7)
Female neonates	9 (52%)	4 (50%)	5 (56%)
Birthweight (g)	3276.0 (323)	3298.1 (326)	3255.6 (339)
Birthweight centile (%)^c^	61 (30)	59 (31)	66 (31)

Data presented as Mean (SD) or n (%)

^a^WHO recommended Asian-specific BMI cut-off that is associated with increased metabolic risk (WHO EC 2004): Normal 18.5–22.9 kg/m^2^, Overweight 23–27.4 kg/m^2^, Obese ≥ 27.5 kg/m^2^

^b^In mid-gestation 75 g three time-point OGTT

^c^By local sex and gestational age-standardized references

### Placenta collection and placental explant culture

Placental explants were cultured as previously described. Briefly, five pieces of villous placental tissue were randomly biopsied from five different sites across the placenta. These biopsies were then cut into small explants (approximately 3 × 3 × 3 mm) and added to each experimental well, with one explant coming from each different placental biopsy.

Explants were cultured in 12 well plates (Thermo-scientific, NunclonTM Delta surface) in basal serum-free CMRL media (1.8 mL, contains a physiological glucose concentration and no detectable fatty acids; GIBCO 1066-L Glutamine, Thermofisher, New York, USA) with 1.5% BSA (to match the albumin: fatty-acid ratio of maternal blood in the last trimester (0.78) (Haggarty et al. 1997); HI Clone fraction V, Culture grade, pH 7.00 lyophilized powder, GE Life Sciences, South Logan, Utah) and either no DHA or ^13^C_22_-DHA (24 µmol/L, 99 atom % ^13^C, 99% CP, Cambridge isotope laboratories), and either no additional (5 mmol/L) or 10 mmol/L final concentration of glucose (Biowest, P5030, France).

The levels of local un-esterified DHA available for placental uptake at the maternal-facing apical membrane of the placental syncytiotrophoblast barrier in-vivo are unknown. Un-esterified DHA was found to be 5.8 (SD 1.6) µmol/L in the maternal vein, 19.3 (2.8) µmol/L in the placental intervillous space containing maternal blood, but the actual amount available at the apical membrane is likely higher since much of the DHA available for uptake is lipid bound and only hydrolysed at the apical membrane surface to release unesterified DHA, which is rapidly internalized (Benassayag et al. 1997). We therefore added 24 µmol/L of ^13^C-DHA in our experiments, which was high enough to enable quantification of a range of ^13^C-DHA placental lipids.

Explants were incubated in a Thermo-scientific Foma Direct heat CO_2_ Incubator, Hepa Class 100 Model 37 °C in a humidified atmosphere of 5% CO_2_/air. All placental processing was finished within 2 h of delivery. Treatments were applied in triplicate wells and explants harvested at 48 h with triplicates combined in a pre-weighed Omnitube. Unlike simple uptake of fatty acids, the conversion of newly taken up ^13^C-DHA into ^13^C-DHA lipids is a relatively slow process such that few ^13^C-DHA lipids could be quantified in experimental times shorter than 48 h (Watkins et al. 2019a). Based on time-course experiments 48 h was selected as optimal for accurate quantification of a wide range of newly synthesized ^13^C-DHA lipids and when structural integrity of placental explants, including the syncytial cell layer, is maintained (Watkins et al. 2019a).

Confirmation of explant viability in these culture conditions were previously assessed by HCG and LDH over the period of culture (Watkins et al. 2019a). Explants were washed with PBS (1 mL) then stored at − 80 ºC until lipid extraction. Lipids were extracted as described in Additional file 2.

### LC–MS/MS methodology

The LCMS method was developed based on our previous method (Watkins et al. 2019a) and the lipidomics methods of the Baker Institute (Weir et al. 2013). A dMRM transition list was developed containing transitions for all lipids possibly containing DHA (22:6). Transitions were then calculated for a ^13^C-DHA (^13^C_22_-DHA) containing partner for each lipid and these candidate transitions added to the method and this method tested on placental lipid extracts from placenta incubated with ^13^C-DHA. Proposed ^13^C-DHA lipid transitions that gave peaks exactly co-eluting with their ^12^C-DHA counterpart were kept, whilst those that did not were removed from the method. Candidate ^13^C-DHA lipid peaks present in lysate from placenta not incubated with ^13^C-DHA were also removed from the list. Additional file 3 illustrates the structure of DHA containing PC 38:6, showing how the incorporation of either ^12^C-DHA or ^13^C-DHA can produce an unlabeled or ^13^C-labeled PC 38:6, respectively, which can be quantified separately by LCMS due to differences in mass, but with identical physical characteristics and elution times. Lipid extracts (5 μL) were injected into an Agilent 6490 triple quadrupole (QQQ) liquid chromatography mass spectrometry (LC–MS/MS) instrument alongside a range of standards and analysed using a targeted dynamic multiple reaction monitoring (dMRM) method as described in Additional file 4.

### Data and statistical analysis

LC–MS/MS data was analysed using Mass Hunter QQQ Quantitative Analysis Version 8 and peak area quantified by integration. Peak areas were normalized against their corresponding lipid class internal standard to give concentration expressed as pmol/ml. Dried placental mass was used to calculate the lipid amount expressed as pmol/mg. Calculations used for analysis are defined in Additional file 4 and quantified lipid data in Additional file 5. Statistical analysis was run on Z-scored log2 lipid amount or enrichment. Linear regression was then run for each lipid (outcome) with each variable of interest (predictor): maternal BMI, fasting glycemia or post-load 2 h glycemia, maternal age, or gestational age at delivery. Sensitivity analyses were performed excluding outliers of fasting glycemia. Linear regression was then run for birthweight centile (outcome) with each lipid (one lipid per model; predictor). Where indicated, multiple linear regression was then performed with mutual adjustments for these variables.

The effects of in-vitro glucose treatment (10 mmol/L) were expressed as log2 fold-change relative to controls from the same placenta with no additional glucose (5 mmol/L). The fold-change was calculated as amount of labeled lipid in explants treated with 10 mmol/L glucose divided by amount of labeled lipid in control explants. Fold-change was then log-2-transformed to give a glucose response value for each lipid. Glucose response therefore represents the relative amount of ^13^C-DHA lipid in placental explants treated with glucose compared to control explants from the same placenta. A ^13^C-DHA lipid glucose response > 0 indicates an increase in ^13^C-DHA lipid compared to the control, while a glucose response < 0 indicates a decrease. Linear regression was used to determine the relationships between glucose response and maternal characteristics or birthweight. Repeated measures analysis of variance was used to test whether glucose treatment altered the relative amount of placental ^13^C-DHA lipid compared to the control and the effects of covariate adjustment with one lipid per model. Data was analyzed in R using packages described in Additional file 4. Additional sensitivity analyses were performed with exclusion of those taking PUFA-containing supplements. For all sets of statistical analyses, the Benjamini–Hochberg method was used to correct for multiple testing to minimize false discovery. Statistical significance was set at a two-sided alpha level of p < 0.05.

## Results

### Placental incorporation of ^13^C-DHA into lipids

**Fig. 1:**
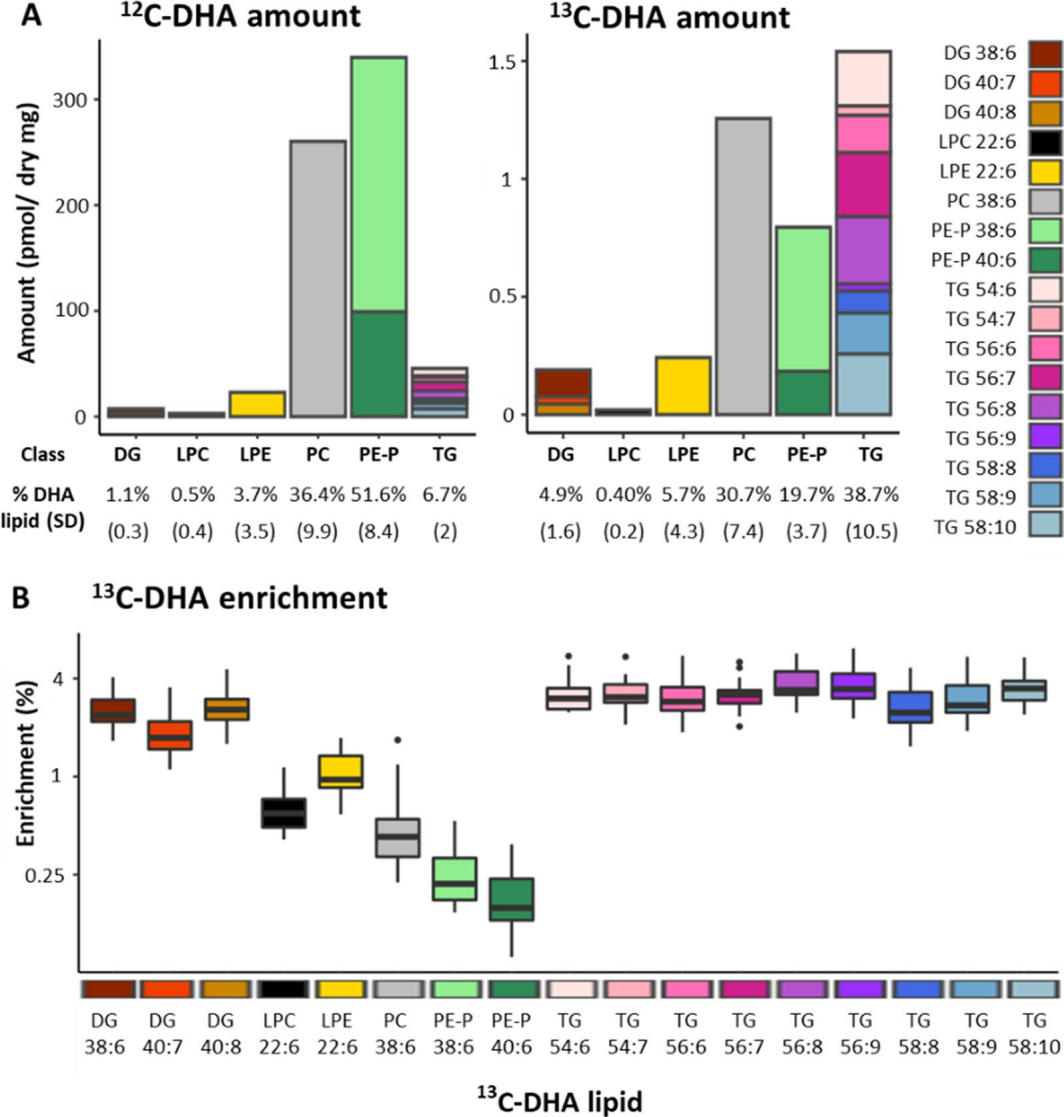
Placental lipids containing ^12^C-DHA and ^13^C-DHA in placental explants from 17 placenta incubated for 48 h with 24 µmol/L ^13^C-DHA at a physiological glucose concentration (5 mmol/L). **A** Amount of each lipid (pmol/mg) with percentages showing relative amount of each lipid class compared to total ^12^C-DHA or ^13^C-DHA lipids. Brackets show standard deviation. Colours represent individual lipids. **B** Boxplots showing the enrichment of each lipid with ^13^C-DHA. Enrichment is the percentage of each lipid containing ^13^C-DHA calculated by the formula 100 * ^13^C-lipid/(^13^C-lipid + ^12^C-lipid). *DHA* docosahexaenoic acid, *DG* diacylglycerol, *LPC* lyso-phosphatidylcholine, *LPE* lyso-phosphatidylethanolamine, *PC* phosphatidylcholine, *PE-P* phosphatidylethanolamine-plasmalogen, *TG* triacylglycerols. Labeled TGs contained ^13^C-DHA and two other fatty acids which cannot be identified with certainty. Since the total number of fatty acid carbons (*x*) and the total number of fatty acid double bonds (*y*) is known for each TG (format *x:y*), the combined carbon count and saturation of the two remaining fatty acids can be calculated, then predicted based on known abundance in humans. Two TGs contained ^13^C-DHA and two saturated lipids (TG 54:6 [predicted 16:0_16:0_22:6], TG 56:6 [predicted 16:0_18:0_22:6]), two contained DHA with one saturated fatty acid and one mono-unsaturated fatty acid (TG 54:7 [predicted 16:0_16:1_22:6], TG 56:7 [predicted 16:0_18:1_22:6 or 16:1_18:0_22:6]) and five TGs contained DHA with unknown combinations of various unsaturated lipids (TG 56:8, TG 58:8, TG 56:9, TG 58:9 and TG 58:10)

Placental explants incubated with stable-isotope ^13^C-DHA for 48 h produced stable-isotope labeled ^13^C-DHA-lipids of which seventeen could be reliably quantified using our LCMS method (Fig. 1). Quantifiable ^13^C-DHA phospholipids and their ^12^C-DHA counterparts included phosphatidyl-ethanolamine-plasmalogens (PE-P 48:6 [16:0_22:6], PE-P 40:6 [18:0_22:6]), phosphatidyl-choline (PC 38:6 [16:0_22:6]), lyso-phosphatidyl-choline (LPC 22:6) and lyso-phosphatidyl-ethanolamine (LPE 22:6). Quantifiable ^13^C-DHA glycerolipids and their ^12^C-DHA counterparts included diacylglycerides (DG 38:6 [16:0_22:6], 40:7 [18:1_22:6], 40:8 [18:2_22:6]) and nine triacylglycerides (TG; all contained ^13^C-DHA and two other fatty acids).

While the bulk of endogenous ^12^C-DHA lipids was found in PE-Ps (51.6% of total ^12^C-DHA lipids ± SD 8.4) followed by PCs (36.4% ± 9.9), then TGs (6.7% ± 2), for freshly produced ^13^C-DHA lipids, most were found in TGs (38.7% of total ^13^C-DHA lipids ± 10.5) and PCs (30.7% ± 7.4) followed by PE-Ps (19.7% ± 3.7) (Fig. 1A). This indicates that newly synthesized total ^13^C-DHA-TGs made up a much greater proportion of newly synthesised DHA lipids than any of the other individual lipid classes. However, the overall total of newly synthesized ^13^C-DHA-phospholipids from all its classes still amounted to more than total ^13^C-DHA-TGs, consistent with previous studies (Johnsen et al. 2009; Gil-Sánchez et al. 2010).

The difference between ^12^C-DHA lipids and their corresponding ^13^C-DHA lipids was especially obvious for PE-P, consistent with the idea that plasmalogens become enriched with PUFA mainly via the slow remodelling of existing phospholipids (Braverman and Moser 2012). Placental PEs (which includes PE-Ps) are known to be an important source of DHA for the fetus (Uhl et al. 2015), while PC 38:6 was previously found to be the most abundant PC with at least 6 double bonds (i.e. possibly DHA containing) in placenta (Uhl et al. 2015), and in placental lipid droplets (Gázquez et al. 2018).

DGs and LPCs made up the lowest proportion of both ^12^C-DHA and ^13^C-DHA labeled lipids, which is expected since these molecules are normally lowly abundant, transitory, and often act as signaling molecules (Wasserman et al. 2020).

Enrichment of each lipid with ^13^C-DHA (i.e. 100*^13^C-lipid/(^13^C-lipid + ^12^C-lipid)) gives an indication of lipid turnover (i.e. how quickly new lipids are produced and how quickly existing lipids are replaced by new lipids) and was found to be highest in TGs and DGs (Fig. 1B). This finding is consistent with the role of DGs as a precursor to most other lipids (Fig. 7) and suggests that TGs provide rapid storage for newly taken up ^13^C-DHA. Phospholipid enrichment in contrast was much lower, consistent with the idea that these structural lipids are produced and replaced more slowly. Similar enrichment patterns of these lipid types had previously been described for placental lipids incorporating ^13^C-PA and ^13^C-OA (Watkins et al. 2019b).

### Association of maternal glycemia and BMI with placental ^13^C-DHA incorporation under culture in a physiological glucose condition (5 mmol/L)

**Fig. 2:**
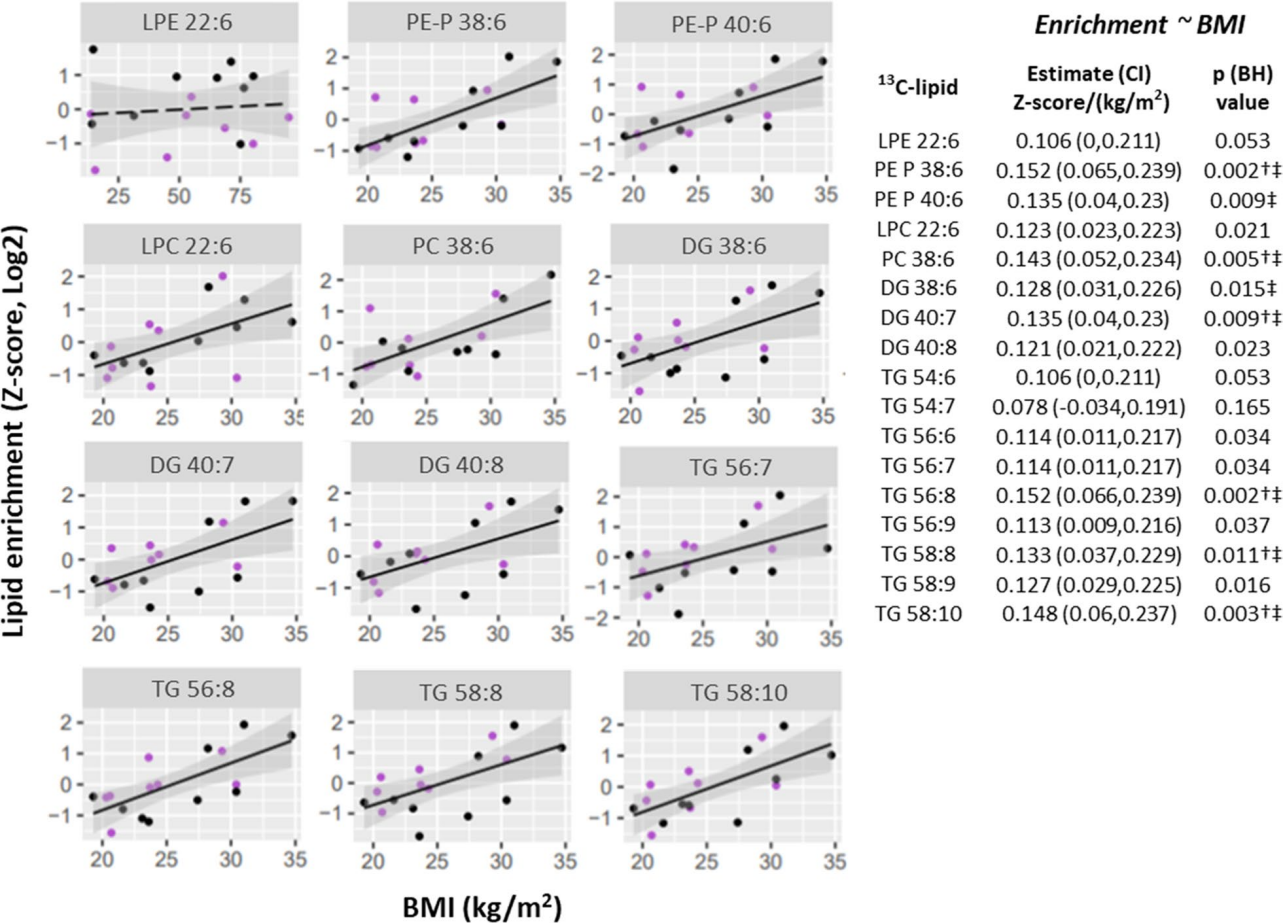
Positive associations of BMI with the *enrichment* of placental ^13^C-DHA lipids (Z-scored, log2 transformed; n = 17 placenta) in culture at a physiological glucose condition (5 mmol/L). Linear regression was run with lipid as the outcome and BMI as the predictor. The Benjamini–Hochberg (BH) method was used to correct for multiple testing. Solid lines show significant associations while dashed lines show non-significant associations. Shaded areas show 95% confidence intervals. Key for dots—Purple: non-GDM, Black: GDM. ^†^Significant after adjusting for fasting glycemia, ^‡^Significant after adjusting for 2 h glycemia. *CI* confidence interval, *DG* diacylglycerol, *DHA* docosahexaenoic acid, *GDM* gestational diabetes, *LPC* lyso-phosphatidylcholine, *LPE* lyso-phosphatidylethanolamine, *PC* phosphatidylcholine, *PE-P* phosphatidylethanolamine-plasmalogen, *TG* triacylglycerol

Maternal BMI was positively associated with placental ^13^C-DHA-lipid enrichment for 14 out of the 17 lipids and associations remained similar after adjusting for maternal fasting or 2 h glycemia (Fig. 2). Of all quantified ^13^C-DHA-lipids, post-load 2 h glycemia was only associated with ^13^C-DHA-LPE enrichment [estimate (95% CI) 0.44 (0.20, 0.68) log2 z-score per mmol/L glucose, p = 0.002; Additional file 6] with associations remaining significant after adjusting for BMI. No association with ^13^C-DHA-lipid enrichment was observed for fasting glycemia, gestational age or maternal age.

**Fig. 3:**
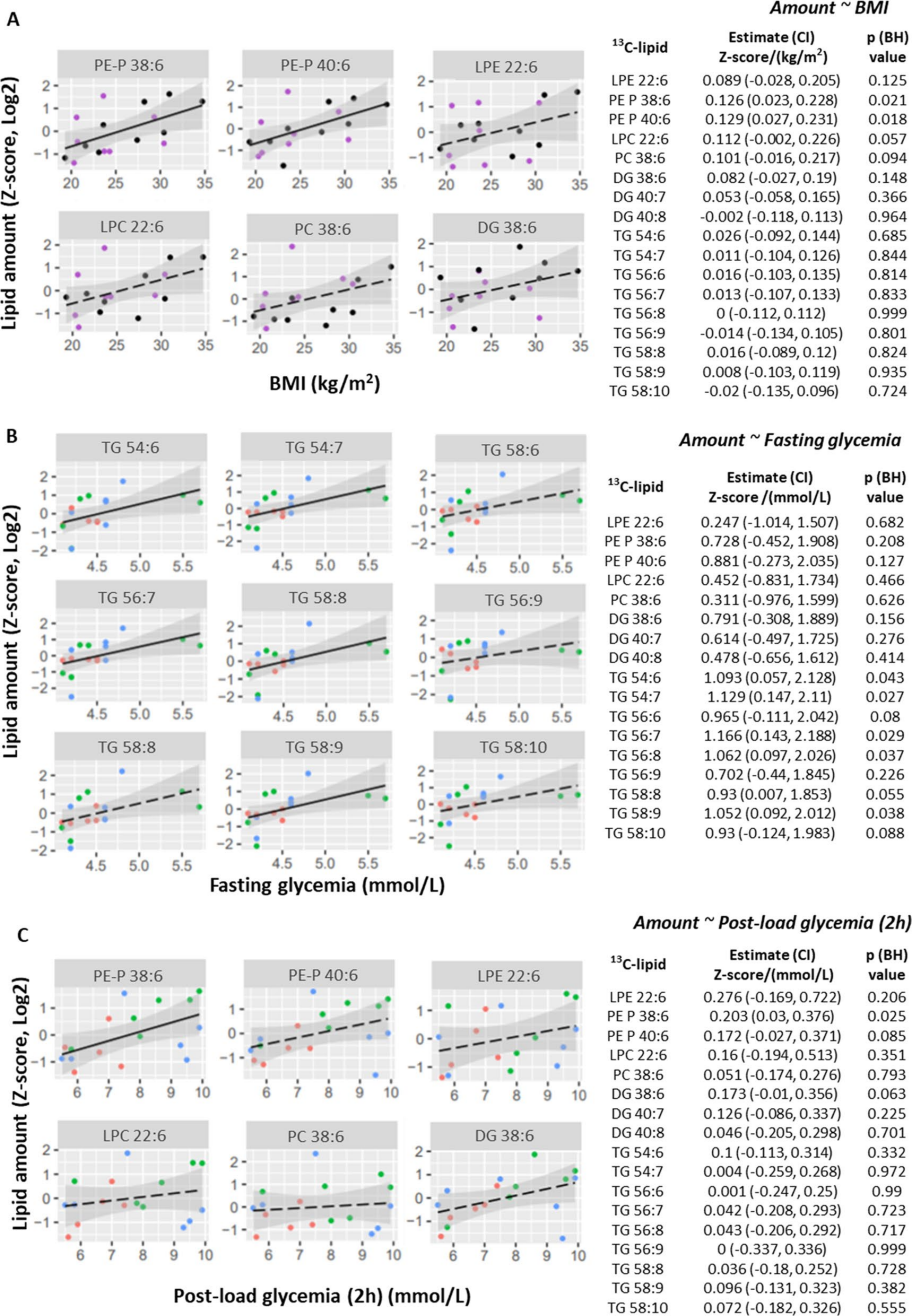
Variations in patterns of association between fasting glycemia (**A**), post-load (2 h) glycemia (**B**) and maternal BMI (**C**) with the *amount* of placental ^13^C-DHA lipids (Z-score, log2; n = 17 placenta). Linear regression was run with lipid as the outcome and glycemia or BMI as the predictor. The Benjamini–Hochberg method was used to correct for multiple testing. Solid lines show statistically significant associations, while dashed lines show non-significant trends. Shaded areas show 95% confidence intervals. Key for dots in **A** and **B**—Red: Normal BMI (< 23 kg/m^2^), Green: Overweight (BMI 23.0 to < 27.5 kg/m^2^), Blue: Obese (BMI ≥ 27.5 kg/m^2^). Key for dots in **C**—Purple: non-GDM, Black: GDM. *BH* Benjamini–Hochberg, *CI* confidence interval, *DG* diacylglycerol, *DHA* docosahexaenoic acid, *GDM* gestational diabetes, *LPC* lyso-phosphatidylcholine, *LPE* lyso-phosphatidylethanolamine, *PC* phosphatidylcholine, *PE-P* phosphatidylethanolamine-plasmalogen, *TG* triacylglycerol

Maternal BMI was also positively associated with ^13^C-DHA phospholipid amount, with significant associations observed for PE-P 38:6 and PE-P 40:6 and similar trends seen for PC 36:6 and LPC 22:6 (Fig. 3A). Post-load 2 h glycemia was also positively associated with ^13^C-DHA phospholipid amount, with significant associations observed for ^13^C-DHA labeled PE-P 38:6 and similar trends observed for PE-P 40:6 and DG 38:6 (Fig. 3C).

Even though BMI was associated with TG enrichment and therefore TG turnover, neither BMI nor post-load glycemia showed any significant associations with ^13^C-DHA TG amount. Instead, antenatal fasting glycemia was positively associated with the amount of ^13^C-DHA labeled TGs (significant for 5 out of the 9 with similar trends seen for the remainder), but not other lipids (Fig. 3B). In sensitivity analyses, which excluded the two outliers of higher fasting glycemia, results remained similar. Similar but non-significant trends in these associations were also observed after mutual adjustment for BMI and fasting glycemia (Additional file 7). Adjustment for maternal age or gestational age at delivery did not materially alter these results. No associations were observed between maternal age or gestational age with any ^13^C-DHA labeled lipid in univariate analyses.

### Association between placental ^13^C-DHA lipid incorporation under culture in a physiological glucose condition (5 mmol/L) and birthweight centile

**Fig. 4:**
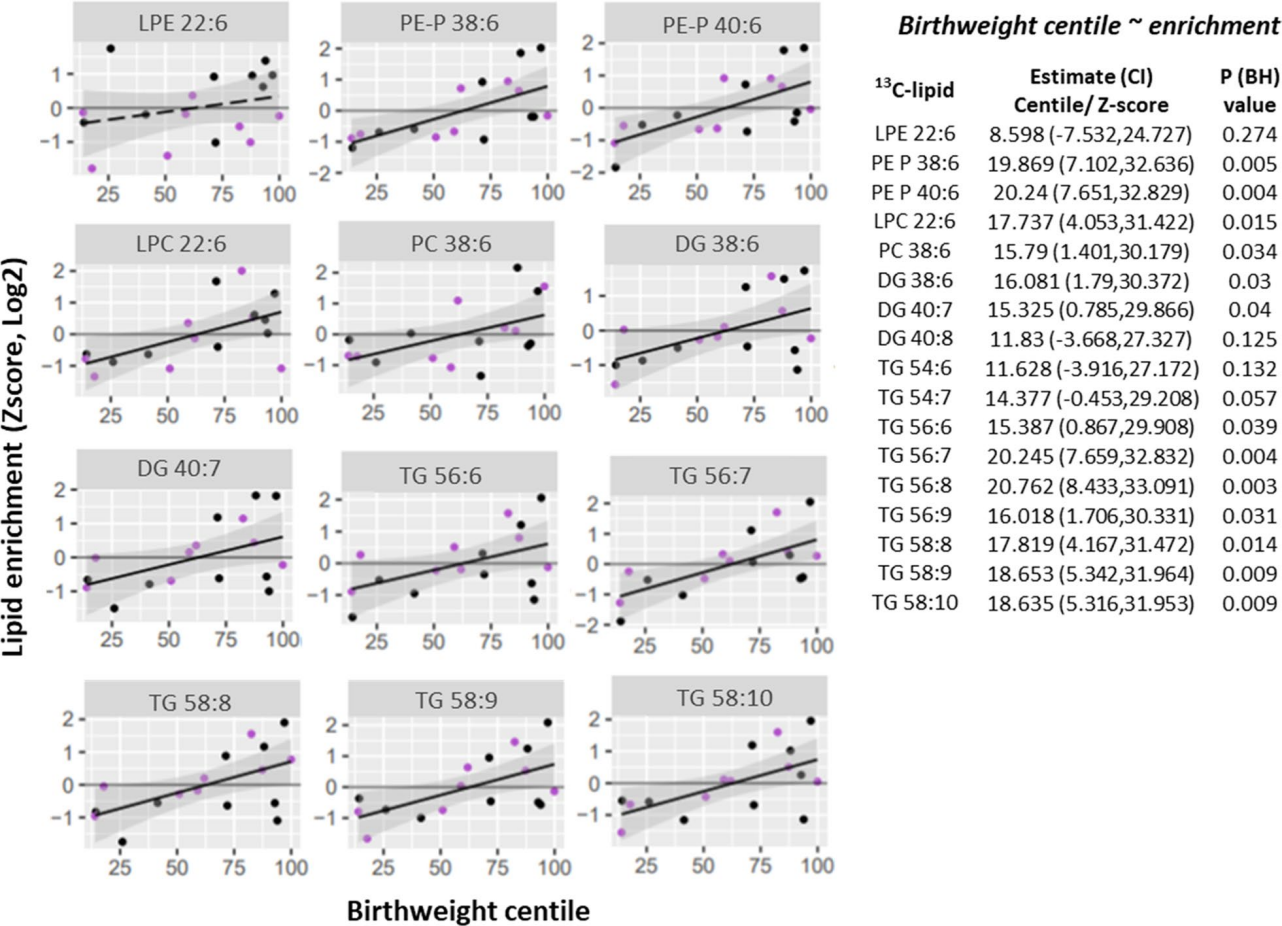
Positive associations between placental ^13^C-DHA-lipid enrichment (Z-score, log2) and birthweight centile. Linear regression was run with birthweight centile as the outcome and either lipid amount or lipid enrichment as the predictor. The Benjamini–Hochberg method was used to correct for multiple testing. Solid lines show significant associations while dashed lines show non-significant results. Shaded areas show 95% confidence intervals. Key—Purple: non-GDM, Black: GDM. *BH* Benjamini–Hochberg, *CI* confidence interval, *DG* diacylglycerol, *DHA* docosahexaenoic acid, *GDM* gestational diabetes, *LPC* lyso-phosphatidylcholine, *LPE* lyso-phosphatidylethanolamine, *PC* phosphatidylcholine, *PE-P* phosphatidylethanolamine-plasmalogen, *TG* triacylglycerol

Birthweight centile was positively associated with increasing ^13^C-DHA enrichment of lipids in general (13 out of 17 ^13^C-DHA lipids, Fig. 4) and increasing amounts of ^13^C-DHA phospholipids (LPC 22:6, PE-P 38:6, PE-P 40:6, Additional file 8).

Since associations were observed between maternal BMI and birthweight centile [estimate (95% CI) 19.9 (7.2, 32.7) % per SD increase in log2-BMI, p = 0.004] and between BMI with DHA-lipids, we hypothesized that BMI may impact birthweight partly through affecting placental DHA metabolism. We thus adjusted for each DHA lipid in turn in our BMI (predictor)-birthweight centile (outcome) model (Additional file 9) to assess for any attenuation of the associations. BMI was no longer significantly associated with birthweight centile after adjusting for the enrichment of DG 38:6, DG 40:7, LPC 22:6, PC 38:6, PE-P 38:6, PE-P 40:6 and 8 out of 9 TGs, or for the amount of LPC 22:6, PE-P 38:6, PE-P 40:6. A reduction in the magnitude of birthweight centile change per unit BMI was also consistently observed across these DHA-lipid models. This suggests that placental DHA lipid metabolism may be one of the mechanistic pathways by which maternal BMI could increase birthweight.

### Glucose treatment in-vitro and placental DHA-lipid metabolism

**Fig. 5:**
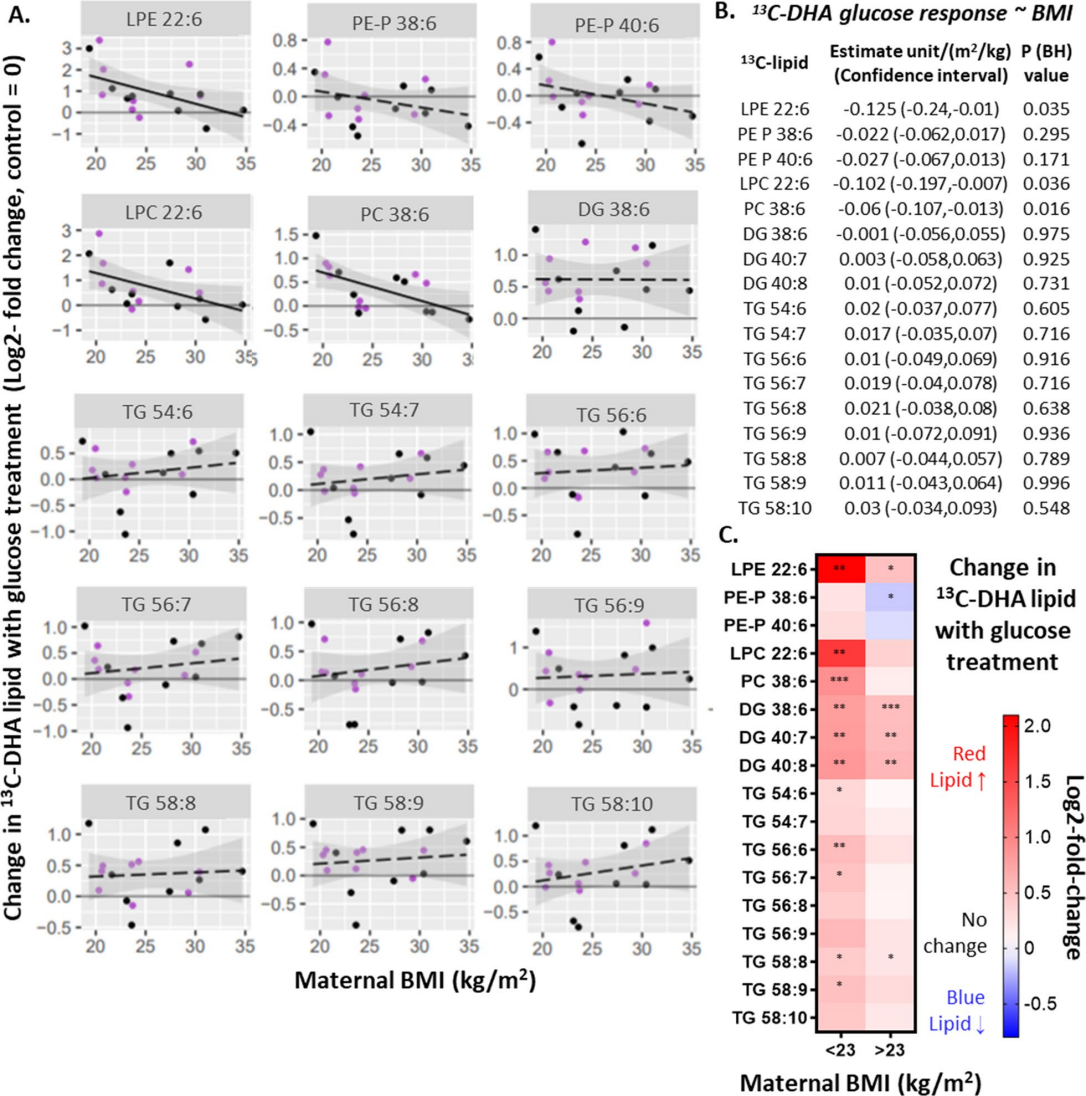
Associations between maternal BMI and change in ^13^C-DHA lipids with glucose treatment (i.e. glucose response, log2-fold change, n = 17). Change in ^13^C-DHA lipid with glucose treatment represents the relative amount of ^13^C-DHA lipid in placental explants treated with glucose (10 mmol/L) compared to control explants (cultured in 5 mmol/L glucose) from the same placenta. Positive values indicate an increase in ^13^C-DHA lipids compared to the control, whilst negative values indicate a decrease. **A**, **B** Linear regression was run with glucose response as the outcome and BMI as the exposure variable. Benjamini–Hochberg (BH) method was used to correct for multiple testing. Solid lines show statistically significant associations, while dashed lines show non-significant results. Shaded areas show 95% confidence intervals. Key for dots—Purple: non-GDM, Black: GDM. **C** Analyses stratified by maternal BMI. Heat map illustrating glucose response in placental explants from normal weight women (BMI < 23 kg/m^2^) and from overweight/obese women (BMI ≥ 23 kg/m^2^). Colours indicate an increase (red; positive glucose response) or a decrease (blue; negative glucose response). Asterisks indicate significant differences between 10 mmol/L glucose treatment and the control (5 mmol/L) *p < 0.05, **p < 0.01, ***p < 0.001. Comparisons were made by repeated measures analysis of variance for each lipid followed by the Benjamini–Hochberg correction for multiplicity. *BH* Benjamini–Hochberg, *CI* confidence interval, *DG* diacylglycerol, *DHA* docosahexaenoic acid, *GDM* gestational diabetes, *LPC* lyso-phosphatidylcholine, *LPE* lyso-phosphatidylethanolamine, *PC* phosphatidylcholine, *PE-P* phosphatidylethanolamine-plasmalogen, *TG* triacylglycerol

To determine if glucose can directly affect placental DHA-lipid metabolism, placental explants were cultured in media with added glucose. Overall, the amount of most ^13^C-DHA lipids in most placental explants was increased by glucose treatment (10 mmol/L) compared with the respective control (5 mmol/L glucose), as indicated by a log2-fold change greater than zero (i.e. dots above the x-axis in Fig. 5). The relative amount of ^13^C-DHA lipids between the glucose-treated and control, for each placenta, is henceforth termed glucose response. Since it has been postulated that how the placenta responds to glucose in-vitro could be dependent on prior exposure to maternal metabolic factors in-vivo, associations between maternal BMI or glycemia, with glucose response were investigated. Neither fasting glycemia nor 2 h glycemia, were associated with glucose response (Table 2). However, BMI was found to be negatively associated with ^13^C-DHA-phospholipid glucose response (Fig. 5).

**Table 2:** Associations between maternal glycemia and change in ^13^C-DHA lipids with in-vitro glucose treatment (glucose response) in placental explants (n = 17)

^13^C-DHA lipid	Fasting glycemia^a^		Post load glycemia (2 h)^a^	
	Estimate (lower CI, upper CI) Relative amount^b^/ (mmol/L)	p value (BH)	Estimate (lower CI, upper CI) Relative amount^b^/ (mmol/L)	p value (BH)
DG 38:6	0.106 (− 0.45, 0.663)	0.911	− 0.091 (− 0.241, 0.059)	0.216
DG 40:7	0.065 (− 0.542, 0.673)	0.822	− 0.108 (− 0.27 ,0.053)	0.172
DG 40:8	0.116 (− 0.506, 0.739)	0.919	− 0.093 (− 0.262, 0.076)	0.261
LPC 22:6	− 0.688 (− 1.732, 0.355)	0.18	− 0.228 (− 0.515, 0.059)	0.111
LPE 22:6	− 0.82 (− 2.092, 0.451)	0.189	− 0.26 (− 0.613, 0.093)	0.137
PC 38:6	− 0.474 (− 0.993, 0.046)	0.071	− 0.111 (− 0.264, 0.041)	0.139
PE-P 38:6	− 0.034 (− 0.45, 0.382)	0.912	− 0.079 (− 0.189, 0.03)	0.186
PE-P 40:6	− 0.196 (− 0.612, 0.219)	0.329	− 0.055 (− 0.172, 0.062)	0.338
TG 54:6	− 0.164 (− 0.741, 0.414)	0.555	− 0.075 (− 0.235, 0.085)	0.334
TG 54:7	− 0.123 (− 0.658, 0.413)	0.632	− 0.059 (− 0.208, 0.09)	0.412
TG 56:6	− 0.243 (− 0.823, 0.337)	0.387	− 0.06 (− 0.225, 0.105)	0.451
TG 56:7	− 0.128 (− 0.729, 0.474)	0.658	− 0.037 (− 0.207, 0.133)	0.65
TG 56:8	− 0.042 (− 0.647, 0.562)	0.883	− 0.053 (− 0.221, 0.115)	0.512
TG 56:9	− 0.256 (− 1.065, 0.553)	0.511	− 0.117 (− 0.34, 0.106)	0.28
TG 58:8	0.112 (− 0.398, 0.622)	0.89	− 0.01 (− 0.155, 0.135)	0.881
TG 58:9	− 0.086 (− 0.628, 0.457)	0.741	− 0.076 (− 0.224, 0.072)	0.289
TG 58:10	0.009 (− 0.649, 0.668)	0.976	− 0.052 (− 0.236, 0.132)	0.553

*CI* confidence interval, *BH* Benjamini–Hochberg corrected

^a^In mid-gestation 75 g three time-point OGTT

^b^Change in ^13^C-DHA lipid with glucose treatment or glucose response (log2-fold-change) represents the relative amount of ^13^C-DHA lipid in placental explants treated with glucose (10 mmol/L) compared to control explants from the same placenta. Positive values indicate an increase in ^13^C-DHA lipids compared to the control, whilst negative values indicate a decrease

Since BMI was associated with phospholipid glucose response, further analyses were conducted with stratification by maternal BMI (normal BMI < 23 kg/m^2^ versus overweight/obese: BMI ≥ 23 kg/m^2^) using the WHO recommended Asian-specific BMI cut-off that is associated with increased metabolic risk (WHO EC 2004). Among cases of normal BMI, glucose treatment (compared with no added glucose control) increased ^13^C-DHA DGs (all three), LPC, LPE, PC and over half of ^13^C-DHA TGs (Fig. 5C). In contrast, in the overweight/obese group glucose treatment (compared with control) increased fewer lipids; there was only increased ^13^C-DHA DGs (all three), LPE and TG 58:8, whilst PE-P 38:6 was decreased in this group (Fig. 5C).

### Glucose-treatment-induced changes in placental DHA metabolism and birthweight

**Fig. 6:**
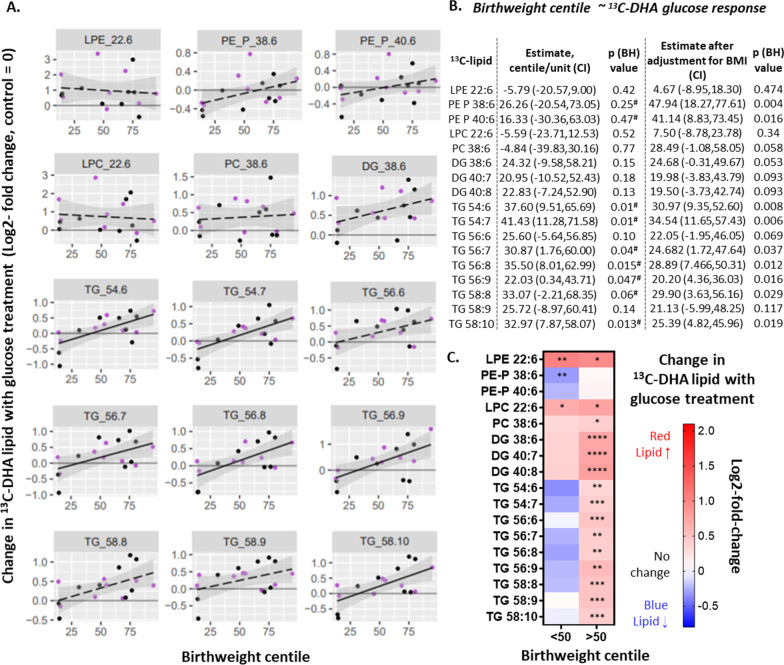
Associations between glucose-treatment-induced changes in ^13^C-DHA lipids (glucose response) and birthweight centile (n = 17). Change in ^13^C-DHA lipid with glucose treatment or glucose response (log2-fold-change) represents the relative amount of ^13^C-DHA lipid in placental explants treated with glucose (10 mmol/L) compared to control explants from the same placenta. Positive values indicate an increase in ^13^C-DHA lipids compared to the control, whilst negative values indicate a decrease. Linear regression was run with birthweight centile as the outcome and glucose response as the exposure variable, with or without adjusting for (Z-score, log-2) BMI. Solid lines show significant associations while dashed lines show non-significant associations. Shaded areas show 95% confidence intervals. Key—Purple: non GDM, Black: GDM. Significance retained after adjusting for (Z-score, log-2) BMI (#). The Benjamini–Hochberg method was used to correct for multiple testing. **C** Heat map illustrating glucose response in placental explants from births with birthweight centile < 50 and from births with birthweight centile > 50. Positive values (red) indicate an increase in ^13^C-DHA lipids compared to the control, while negative values (blue) indicate a decrease. Asterisks indicate significant differences between 10 mmol/L glucose treatment and the control (5 mmol/L) *p < 0.05, **p < 0.01, ***p < 0.001, ****p < 0.0001. Comparisons were made for each lipid by repeated measures analysis of variance followed by the Benjamini–Hochberg correction for multiple comparisons. *BH* Benjamini–Hochberg, *CI* confidence interval, *DG* diacylglycerol, *DHA* docosahexaenoic acid, *GDM* gestational diabetes, *LPC* lyso-phosphatidylcholine, *LPE* lyso-phosphatidylethanolamine, *PC* phosphatidylcholine, *PE-P* phosphatidylethanolamine-plasmalogen, *TG* triacylglycerol

Next, we examined the association between glucose response and birthweight, since how the placenta responds to glucose during pregnancy might impact fetal growth. Positive associations were found between the glucose response of ^13^C-DHA-TGs and birthweight centile (six out of nine TGs; Fig. 6), and these associations remained similar after adjusting for maternal BMI (Fig. 6), suggesting that BMI does not substantially confound this relationship with DHA-TGs. Similar trends were seen between the glucose response of DGs and PE-Ps and birthweight centile. Moreover, the association of the glucose response of ^13^C-DHA PE-P 38:6 and PE-P 40:6 with birthweight centile was even strengthened and became statistically significant after adjusting for BMI [estimate (95% CI) 47.9 (18.3, 77.6) % per increase in log2-fold-change, p = 0.004; and 41.1 (8.8, 73.4), p = 0.016, respectively].

Since glucose response was associated with birthweight centile, with decreases generally seen at lower birthweight centiles, and increases generally seen with higher birthweight centiles, cases were stratified by birthweight 50th centile for further analyses to better identify patterns of glucose-treatment-induced DHA-lipid changes. Among placentas of heavier neonates (birthweight centile > 50%) there were increases in almost all ^13^C-DHA lipids (except for the PE-Ps) with glucose treatment (compared with no added glucose control), but among placentas of lighter neonates (birthweight centile < 50%) there were fewer ^13^C-DHA-lipid alterations with increases only seen in lyso-phospholipids (^13^C-DHA-LPC and ^13^C-DHA-LPE), while PE-P 38:6 was decreased in this group (Fig. 6C).

With the exclusion of cases where antenatal PUFA-containing supplements were taken (leaving n = 11 for analysis), the magnitude and the direction of all reported associations were materially unchanged (data not shown) compared with results from the entire group of 17 cases.

## Discussion

Findings here are consistent with our hypothesis that the activity of term placental DHA-lipid synthesis is increased by the programing effects of higher maternal BMI and higher glycemia, and that this may in turn result in increased birthweight. Higher maternal BMI was associated with greater placental enrichment in ^13^C-DHA DGs, TGs and phospholipids, and greater phospholipid amounts, while fasting glycemia was associated with increases in the amount of ^13^C-DHA-TGs, suggesting that these maternal characteristics affect different DHA metabolic pathways in placenta. Furthermore, positive associations between newly synthesized ^13^C-DHA-lipids and birthweight suggest that placental DHA-lipid metabolism during pregnancy could influence fetal growth. Glucose treatment in-vitro at 10 mmol/L, equivalent to excursions seen in diabetes, increased the amount of almost all newly synthesized ^13^C-DHA lipids, but the magnitude of glucose-treatment-induced ^13^C-DHA phospholipid increases were reduced with increasing maternal BMI. Taken together, increasing maternal BMI programs the placenta to increase DHA-lipid synthetic capacity, which is associated with increasing birthweight, but at the same time BMI may limit the stimulatory effect of glucose on placental DHA-phospholipid synthesis. We therefore postulate that the placental-DHA-lipid-mediated actions leading to an increase in birthweight with increasing maternal glycemia could be altered in the possible co-morbid condition of higher BMI.

### Associations between maternal BMI or glycemia with DHA lipid metabolism

Being able to reliably measure the incorporation of ^13^C-DHA into 17 newly synthesized ^13^C-DHA lipids was an improvement over our previous DHA methods that were only able to measure three ^13^C-DHA TGs (Watkins et al. 2019b). Further, we confirmed our previous findings that programed differences in placental lipid metabolism by maternal BMI and glycemia are retained by placental explants in culture (Watkins et al. 2019a).

**Fig. 7:**
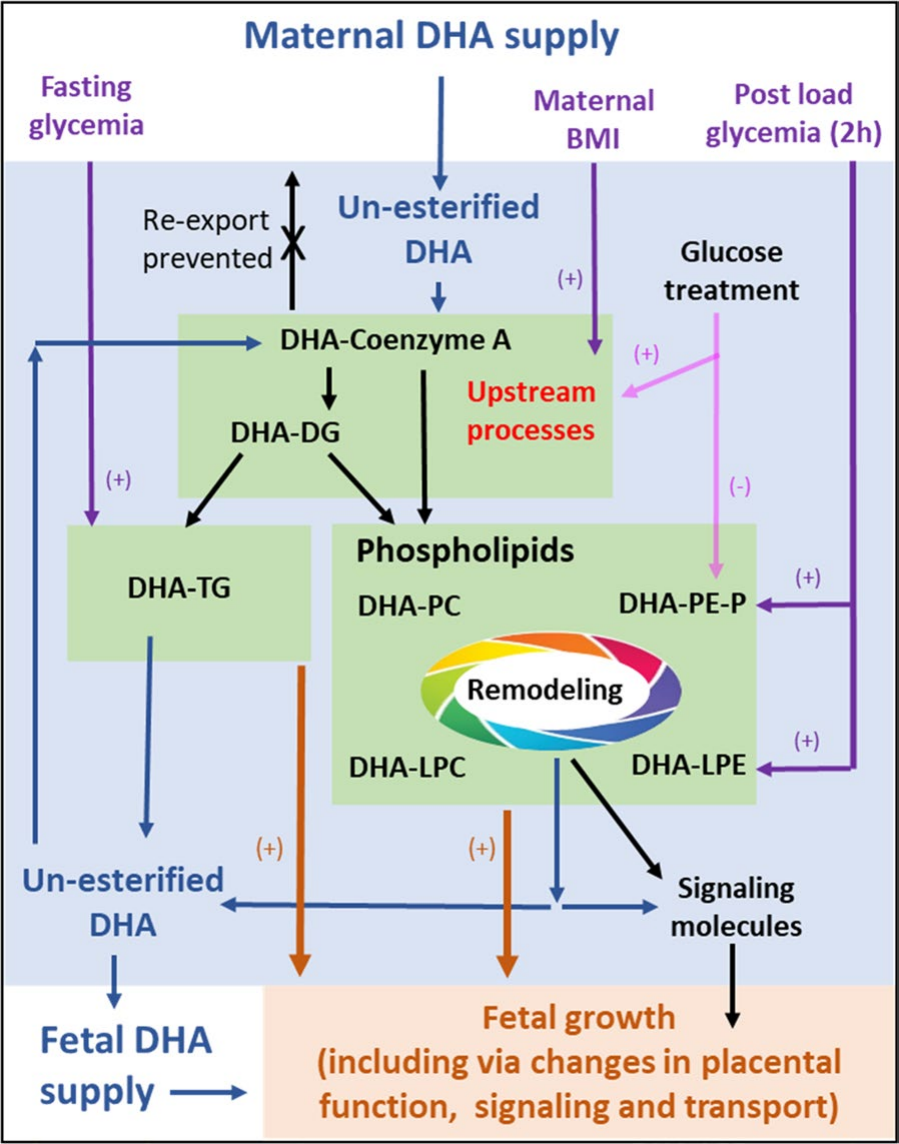
Placental DHA metabolism regulates the amount of DHA available for the synthesis of signaling molecules and for fetal supply. The conversion of DHA into DHA-COA and lipids such as diacylglycerols (DG), triacylglycerols (TG), or phospholipids such as phosphatidylcholine (PC) or phosphatidylethanolamine-plasmalogen (PE-P) traps DHA in the placenta and prevents re-export. DHA released from lipids (blue arrows), may be remade into lipids, used to form signaling molecules such as eicosanoids or exported to the fetus. In our experiments, increased BMI (purple) and in-vitro glucose treatment (pink) was associated with the increased synthesis of most ^13^C-DHA-lipids, suggesting that upstream processes such as activation into DHA-CoA or the early stages of lipid synthesis may be affected. Glucose treatment also decreased PE-Ps suggesting a separate PE-P specific process may also be affected. Fasting glycemia (purple) is only associated with TGs, suggesting a link with TG specific processes. DHA lipid metabolism and change in response to glucose treatment, was also associated with birthweight centile (shown by brown arrows) suggesting that placental DHA metabolism could influence birthweight

The association of maternal BMI and short term in-vitro glucose treatment with almost all ^13^C-DHA lipids, including DHA-DGs, a precursor of other DHA-lipids in placental explants, suggests that these factors influence common upstream processes (Fig. 7) which may include activation into DHA-COA (by placental acyl-CoA synthetase long-chain [ACSL] 2, 3 and 4) or the early stages of lipid synthesis, or placental DHA import and intracellular transport by enzymes such as fatty acid transport proteins (FATP and CD36) and fatty acid binding protein (FABP), which are known to be increased in GDM and obesity (Magnusson et al. 2004; Radaelli et al. 2009; Díaz et al. 2015; Hall et al. 2003; Segura et al. 2017). Whereas the more chronic exposure to antenatal fasting glycemia being only associated with TGs, while post-load glycemia associated mostly with PE-P and LPE, suggest differential glycemic impact (directly or indirectly) on specific downstream lipid metabolic pathways. That in-vitro glucose treatment also decreased ^13^C-DHA PE-Ps, a change in the opposite direction to all other lipids, suggests that glucose particularly influences PE-P specific downstream process. Overall glucose appears to influence DHA processing at multiple points of the various DHA-lipid metabolic pathways (Fig. 7).

At high BMI, there appeared to be limited capacity for further glucose-induced increases in DHA-lipid synthesis in an already heightened DHA-lipid state, but there was still capacity for glucose-induced decreases, for example, in ^13^C-DHA-PE-P. The less exaggerated glucose-induced increase in placental DHA-phospholipids was only observed with increasing maternal BMI, but not by prior in-vivo glycemia exposure, suggesting that BMI (and not maternal glycemia) specifically induces a lasting programing effect on the glucose-responsive aspects of placental DHA-metabolism. Possibly, the critical window period for such programming occurs early in gestation, when the placenta is exposed to pre-pregnancy factors like high BMI, predating the development of gestational hyperglycemia, which typically arises from mid-gestation onwards. Since our study did not include cases of pre-existing diabetes, the potential programming effect of more established hyperglycemia periconception and during very early gestation could not be determined.

In agreement with our current work, several studies had reported increased placental TGs and lipid droplets with maternal hyperglycemia and in-vitro glucose treatment (Visiedo et al. 2015; Visiedo et al. 2013; Mishra et al. 2020; Tewari et al. 2011), while others showed that obesity is linked with increases in many placental lipid classes (Calabuig-Navarro et al. 2017). However, these studies did not specifically measure the DHA content of lipids. Studies that measured naturally occurring DHA lipids found placental DHA-phospholipids to be elevated in GDM or obesity (Uhl et al. 2015; Bitsanis et al. 2006), consistent with our in-vitro ^13^C-DHA studies. However, these studies cannot separate the systemic effects of maternal and fetal DHA metabolism and availability, from local placental processing. Our study, in contrast, demonstrated that both maternal BMI and glycemia influence placental DHA lipid metabolism itself, and that different lipid pathways are affected by BMI and glycemia.

### Placental DHA uptake, metabolism and export

Studies on the in-vivo processing of orally-administered ^13^C-DHA in an obese pregnant population reported reduced placental ^13^C-DHA phospholipids but not TGs with GDM, possibly as a consequence of altered maternal metabolism and placental uptake (Pagán et al. 2013). However, this study also reported that it was the cord/maternal plasma ratio, rather than the placenta/maternal ^13^C-DHA plasma ratio that was most decreased in GDM, suggesting that fetal DHA deficiency is a consequence of alterations in both placental DHA uptake and metabolism (Pagán et al. 2013). The phospholipids involved were not identified, but we observed a similar decrease in freshly produced ^13^C-DHA PE-Ps (a major class of DHA-containing phospholipids) with glucose treatment in the high BMI population.

Isolated primary human trophoblasts from GDM pregnancies were reported to take up less ^14^C-DHA compared to controls over a short incubation of 2 h and that uptake was not affected by in-vitro glucose treatment (Araújo et al. 2013). This may appear inconsistent with our findings of increased ^13^C-DHA-lipids with increasing glycemia and with in-vitro glucose treatment of placental explants. However, measurable incorporation of ^13^C-labeled fatty acids into lipids by placental explants takes more than 3 h (Watkins et al. 2019a), thus the published experiments likely measured (by total radioactivity) predominantly uptake of un-esterified DHA rather than incorporation into lipids like in our experiments.

If indeed DHA uptake is reduced in GDM, our finding of increased placental ^13^C-DHA-lipids (particularly DHA-TGs that are quickly synthesized and turned-over to prevent re-export) with glycemia and glucose treatment*,* suggests that the hyperglycemia-exposed placenta may compensate for reduced uptake by increasing DHA trapping in placenta and DHA-lipid synthesis. Although such compensation mechanisms may protect the placenta from DHA deficiency, they may not necessarily increase fetal DHA supply. Indeed, it has been suggested that increased placental DHA-lipid content associated with hyperglycemia or BMI represents increased placental retention and decreased export of un-esterified DHA to the fetus (Uhl et al. 2015; Bitsanis et al. 2006; Mishra et al. 2020). Unfortunately, our understanding remains limited as studies have not assessed fetal tissue DHA utilization as a determinant of cord blood DHA levels.

### DHA placental metabolism and birthweight

Variations in placental DHA-lipid metabolism could influence fetal growth and development, through providing DHA-lipid substrates for growth, and by regulating the synthesis and release of DHA-lipid signals to affect local placental function and fetal nutritional supply, as well as maternal and fetal metabolism in distant tissues (Haggarty 2002; Lager and Powell 2012; Herrera and Ortega-Senovilla 2018b). Whether the positive association between fresh placental ^13^C-DHA lipids with birthweight in our study imply a causal relationship remains unclear. Nonetheless, our findings are consistent with studies describing increased placental DHA-neutral lipids (TGs and cholesterol esters) with higher neonatal body fat (Varastehpour et al. 2006), and more placental phospholipid fraction DHA from a normal birthweight group compared with a low birthweight group, possibly mediated by DHA-induced PPARγ-promotion of fetal growth (Meher et al. 2016). In general, lyso-phospholipids are known to regulate a wide array of metabolic processes, including insulin-induced release of glycogen and lipid synthesis, and mitochondrial function (Soga et al. 2005; Hollie et al. 2014; Labonté et al. 2010; Rivera and Chun 2006). Much of the cord blood lyso-phospholipids may originate from the placenta (Gázquez et al. 2018; Ferchaud-Roucher et al. 2019) and cord blood lyso-phospholipids, including DHA-LPC (Hellmuth et al. 2017) as well as cord DHA phospholipids in general (Foreman-van Drongelen et al. 1995; Felton et al. 1994), have been positively associated with birthweight. Increased placental DHA lyso-phospholipids (and their phospholipid precursors) observed with higher maternal BMI, post-load glycemia and in heavier neonates in our study, could possibly result in the transfer of more lyso-phospholipids to the fetus and increase fetal growth. Furthermore the activation of placental secretory phospho-lipase A2 (which produces lyso-phospholipids from placental phospholipids) is associated with higher neonatal adiposity (Varastehpour et al. 2006).

Maternal glycemia is known to increase birthweight largely mediated through transplacental glucose supply. Our findings of in-vitro glucose-induced increases in most ^13^C-DHA lipids in placentas from heavier neonates, but only increased ^13^C-DHA lyso-phospholipids in placentas from lighter neonates, suggests that variations in glucose-responsive placental DHA lipid metabolism in-vivo may also partly explain glycemia-promoted fetal growth. Our finding that the magnitude of association between BMI and birthweight centile was reduced following adjustment for individual ^13^C-DHA lipids raises the possibility that placental DHA metabolism could also partially mediate the birthweight-promoting effects of maternal BMI. However, much larger studies will be needed to confirm the mediation effects of DHA-lipids.

Our data showed positive associations between DHA-PE-Ps and birthweight centile, suggesting that PE-P may promote fetal growth, possibly by acting as a large DHA reservoir for fetal supply and synthesis of signaling molecules. However, in contrast to other lipids, in-vitro glucose treatment decreased placental ^13^C-DHA-PE-P, especially with maternal obesity and in cases of lighter neonates. Decreased PE-P may reflect decreased production (thus reducing DHA storage) and/or increased catabolism (which would release un-esterified DHA). We speculate that glucose-induced reduction of placental DHA-PE-P when maternal BMI is high may enable some neonates to compensate for glucose-induced growth acceleration, resulting in more moderated birthweights and lower risk of macrosomia, but possibly at the expense of fetal DHA supply, which is thought to be disrupted in GDM and maternal obesity. Regulation of placental DHA-PE-P metabolism appears pivotal to such a balancing act and should be a focus of future research in pregnancies complicated by diabetes and obesity.

Alterations in placental DHA metabolism could change the availability of un-esterified DHA, DHA-lipids and various DHA-based signaling molecules which could influence fetal growth in different and divergent ways (Hellmuth et al. 2017); the relative strength of influence between them remains undetermined. Our findings of increased DHA-lipids in placenta with maternal metabolic disorders that promote fetal growth, may represent placental retention of some DHA-lipids or increased bioavailability of specific DHA-lipids for fetal transfer and regulation of placental function. These concepts are in keeping with studies reporting an overall lower total DHA content in the fetal circulation with maternal metabolic disorders, since circulating DHA lipids reflect the combined impact of variations in placental metabolism and transplacental transfer, as well as fetal tissue consumption and metabolism, which are likely to increase with greater fetal size. Whether increased placental DHA lipids underlie part of the pathophysiology of excessive fetal growth associated with GDM and maternal obesity (which is more in keeping with our findings that adjustment for DHA-lipids attenuated associations between maternal BMI and birthweight) or could actually be an adaptive response to limit the impact of the maternal condition on the offspring through placental lipid retention remains inconclusive. We also do not exclude the possibility of reverse causation where increased birthweight may be driving increased placental DHA metabolism as part of fetal-tailoring of its own nutritional supply.

### Limitations of the study

The modest sample size in this in-vitro study limits the ability to study interactions between different clinical factors, and other demographic factors such as ethnicity and sex, and we are therefore unable to definitively determine whether DHA-lipids mediate links between maternal BMI or glycemia with birthweight. Consideration of the potential effects of pre-pregnancy BMI and total gestational weight gain were not possible due to the lack of data while noting that a measured weight in early pregnancy provides a more precise assessment of pre-pregnancy weight than recalled weight (Inskip 2020). Since participants were Chinese and Indian, findings may not be generalizable to other ethnicities. A balance of GDM/non-GDM and high/normal BMI cases were selected to understand mechanisms across a spectrum without the extremes of hyperglycemia consistent with pre-existing type 1 and 2 diabetes, and of morbid obesity (BMI > 40 kg/m^2^), and therefore may not represent wider variation within the general pregnant population. Our method does not assume that the syncytiotrophoblast is the only cell type involved in the regulation of the transplacental transfer of DHA. Using mixed cell placental explants has the advantage of preserving microarchitecture and maintenance of cell–cell interactions, including lipid signaling, which may participate in the regulation of overall placental DHA metabolism. However, our method precludes the ability to pinpoint the actual cell types within which newly produced DHA-lipids are synthesized and stored. Placental explants were cultured under 20% O_2_ tension, which is higher than the 8% O_2_ tension within the placental bed and effects may be different in-vivo. The incorporation of ^13^C-DHA into other lipids not discussed here could not be reliably quantified using this methodology. Our method does not allow the quantification of transfer of DHA from the maternal to fetal compartments and exactly how placental metabolism may alter the availability of DHA, either as un-esterified fatty acids or in other lipid forms, for transfer to the fetus is still unclear.

## Conclusions

Our novel evidence demonstrate that maternal BMI and glycemia could increase placental DHA-lipid synthesis itself, without mediation by other confounding factors such as maternal lipid supply and placental blood flow. Increases in placental DHA-lipid production could be one potential mechanism by which higher maternal BMI and glycemia could lead to increased birthweight. Maternal BMI, fasting and post-load glycemia differentially influenced the placental capacity to synthesize individual DHA-lipids. Furthermore, glucose-responsive placental DHA-lipid metabolism can be programed by maternal BMI to limit glucose-stimulation of excessive placental DHA-lipid synthesis and turnover, and may represent a compensatory mechanism to moderate glucose-driven acceleration in fetal growth, but possibly at the expense of transplacental fetal DHA supply. Future studies with larger sample sizes will be required to confirm these postulations and pave the way to development of different approaches to intervention that are tailored to maternal BMI and glycemia status to optimize fetal DHA supply and growth.

## Supplementary Information

**Additional file 1. **Clinical characteristics.

**Additional file 2. **Lipid extraction.

**Additional file 3. **Structure of PC 38:6 illustrating the incorporation of ^13^C-DHA or ^12^C-DHA (A) and chromatogram illustrating the identical retention times of ^13^C and ^12^C PC 38:6 by LCMS (B).

**Additional file 4. **LCMS Methodology.

**Additional file 5. **Lipid data.

**Additional file 6. **The association of post-load glycemia with the *enrichment* of placental ^13^C-DHA lipids (Z-score, log2 transformed) for 17 placenta.

**Additional file 7. **Associations (linear regression) between maternal metabolic factors and lipid amount (Z-score, log2) after mutual adjustment

**Additional file 8. **The association of ^13^C-DHA-lipid amount (Z-score, Log2) with birthweight centile for 17 placenta.

**Additional file 9. **Association between BMI (Z-score, log2) and birthweight centile adjusted for each DHA lipid.

## Data Availability

The dataset supporting the conclusions of this article are included within the article and its additional files.

## Abbreviations

BMI: Body mass index
BSA: Bovine serum albumin
CMRL: Connaught Medical Research Laboratories
DG: Diacylglycerols
DHA: Docosahexaenoic acid
dMRM: Dynamic multiple reaction monitoring
GDM: Gestational diabetes mellitus
IS: Internal standard
LC–MS/MS: Tandem liquid chromatography mass spectrometry
LC-PUFA: Long-chain polyunsaturated fatty acid
LPC: Lyso-phosphatidylcholine
LPE: Lyso-phosphatidylethanolamine
PC: Phosphatidylcholine
PE: Phosphatidylethanolamine
PE-P: Phosphatidylethanolamine plasmalogen
PUFA: Polyunsaturated fatty acids
TG: Triacylglycerol
