# Supplementary material for: Placental 13C-DHA metabolism and relationship with maternal BMI, glycemia and birthweight
Source: Mol Med. 2021 Aug 6;27:84. doi: 10.1186/s10020-021-00344-w (PMC8349043; doi:10.1186/s10020-021-00344-w)
Supplement: Supplementary file 1 — Additional file 1. Clinical characteristics. [file 10020_2021_344_MOESM1_ESM.docx]

**Additional file 1**

| **Clinical characteristics** | | | | | | | | | | |
| --- | --- | --- | --- | --- | --- | --- | --- | --- | --- | --- |
| **Code** | **GDM status*** | **Glycemia fasting (mmol/L)** | **Glycemia 2h**  **(mmol/L)** | **Maternal Ethnicity** | **Maternal Age (years)** | **Maternal BMI**  **(kg/m^2^)** | **Birth weight (g)** | **Gestational age (days)** | **Birth weight centile** | **Neonate Sex** |
| PG_3 | Normal | 4.6 | 5.8 | Indian | 32 | 24.3 | 3455 | 280 | 58.9 | M |
| PG_4 | GDM | 4.1 | 7.4 | Indian | 36 | 19.3 | 3510 | 277 | 72.0 | M |
| PG_5 | Normal | 4.4 | 7.8 | Indian | 31 | 29.3 | 3365 | 267 | 82.5 | M |
| PG_6 | Normal | 4.8 | 7.5 | Chinese | 30 | 23.6 | 3385 | 270 | 87.3 | F |
| PG_7 | Normal | 4.2 | 5.5 | Chinese | 34 | 23.7 | 2890 | 276 | 17.8 | F |
| PG_8 | Normal | 4.5 | 5.9 | Chinese | 33 | 20.3 | 3210 | 277 | 50.9 | F |
| PG_9 | Normal | 4.4 | 7.0 | Chinese | 31 | 20.6 | 3450 | 284 | 61.9 | F |
| PG_10 | GDM | 4.5 | 6.7 | Chinese | 30 | 21.6 | 2880 | 266 | 41.4 | F |
| PG_11 | Normal | 4.2 | 5.8 | Chinese | 38 | 30.4 | 3820 | 261 | 99.9 | M |
| PG_13 | GDM | 4.3 | 8.6 | Indian | 35 | 28.2 | 3185 | 269 | 71.3 | F |
| PG_15 | GDM | 4.1 | 9.6 | Indian | 29 | 34.7 | 3315 | 267 | 88.1 | F |
| PG_16 | GDM | 5.7 | 8.0 | Indian | 35 | 30.4 | 3590 | 274 | 92.8 | F |
| PG_18 | GDM | 4.6 | 9.9 | Chinese | 35 | 23.6 | 2885 | 268 | 26.0 | M |
| PG_19 | GDM | 5.5 | 9.9 | Chinese | 39 | 31.0 | 3635 | 271 | 97.0 | F |
| PG_20 | GDM | 4.2 | 9.5 | Chinese | 36 | 23.1 | 2770 | 269 | 14.3 | M |
| PG_21 | Normal | 4.2 | 5.6 | Chinese | 31 | 20.7 | 2810 | 271 | 14.0 | M |
| PG_22 | GDM | 4.6 | 9.3 | Chinese | 32 | 27.4 | 3530 | 266 | 93.9 | M |

*Universal screening for gestational diabetes (GDM) was performed at mid-gestation by a three time-point 75 g oral glucose tolerance test (OGTT) using WHO 2013 criteria.
