# Supplementary material for: Placental 13C-DHA metabolism and relationship with maternal BMI, glycemia and birthweight
Source: Mol Med. 2021 Aug 6;27:84. doi: 10.1186/s10020-021-00344-w (PMC8349043; doi:10.1186/s10020-021-00344-w)
Supplement: Supplementary file 2 — Additional file 2. Lipid extraction. [file 10020_2021_344_MOESM2_ESM.docx]

**Additional file 2. Lipid extraction**

Frozen placental explants were freeze-dried, weighed, and then lysed using a bead-ruptor homogeniser and phosphate buffered saline (PBS, 1200 μl). Lysate (40 µl) was transferred to an Eppendorf containing 800 µL Butanol/Methanol (1:1) and 10 µl Internal standard mix (Additional file 4E). All solvents were LCMS grade and were purchased from Merck or Thermo-fisher scientific (iso-propyl-alcohol). Samples were briefly vortexed, then sonicated for 30 minutes in an ice bath, then shaken for a further 30 minutes at 4 °C. Samples were then centrifuged at 13,000 rpm for 10 minutes, the supernatant transferred to a HPLC tube (La-Pha-Pack, Germany) and stored at -80 °C. Five quality control samples (BQC) and two blanks were extracted with every placenta. Quality control samples (BQC) were made from placenta lysate pooled from multiple participants, aliquoted and stored at -80 °C.
