# Supplementary material for: Placental 13C-DHA metabolism and relationship with maternal BMI, glycemia and birthweight
Source: Mol Med. 2021 Aug 6;27:84. doi: 10.1186/s10020-021-00344-w (PMC8349043; doi:10.1186/s10020-021-00344-w)
Supplement: Supplementary file 3 — Additional file 3. Structure of PC 38:6 illustrating the incorporation of 13C-DHA or 12C-DHA (A) and chromatogram illustrating the identical retention times of 13C and 12C PC 38:6 by LCMS (B). [file 10020_2021_344_MOESM3_ESM.docx]

**Additional file 3**


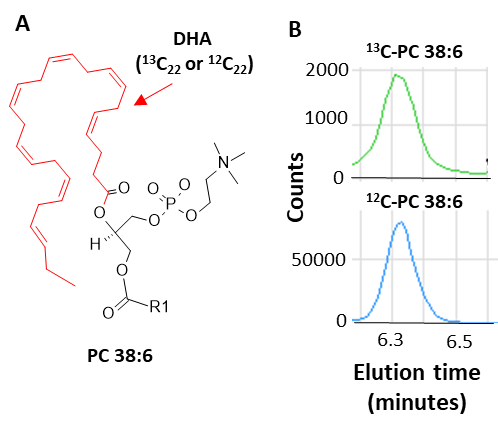


Additional file 3. Structure of PC 38:6 illustrating the incorporation of ^13^C-DHA or ^12^C-DHA (A) and chromatogram illustrating the identical retention times of ^13^C and ^12^C PC 38:6 by LCMS (B).
