# Supplementary material for: Placental 13C-DHA metabolism and relationship with maternal BMI, glycemia and birthweight
Source: Mol Med. 2021 Aug 6;27:84. doi: 10.1186/s10020-021-00344-w (PMC8349043; doi:10.1186/s10020-021-00344-w)
Supplement: Supplementary file 4 — Additional file 4. LCMS Methodology. [file 10020_2021_344_MOESM4_ESM.docx]

**Additional file 4. LCMS Methodology**

**Additional file 4A**: Chromatography was performed using a 2.1 x 100 mm 1.8 μm Zorbax Eclipse Plus C18 RRHD (Agilent Technologies) column and the gradient and LCMS conditions described in Additional file3E. Lipids incorporating one ^13^C_22_-DHA showed *m/z* values that were larger by 22 mass units compared with their respective ^12^C_22_-DHA endogenous counterparts but displayed identical elution times. Samples were randomly analyzed with batch quality control samples (BQC) and blanks measured at regular intervals. ^12^C-DHA lipids were considered quantifiable if BQC percent standard deviation for that lipid was less than 25% and peak area was at least 10x that of the batch blank. ^13^C-DHA lipids were considered quantifiable if the peak co-eluted with a well quantified ^12^C-DHA counterpart, and if the peak area was at least 10x that of the batch blank and at least 3x that of placental explant lysate not incubated with ^13^C-DHA. Lipids with poor quality peak shape were excluded.

**Additional file 4B:** Mobile phase A: 50% water, 30% acetonitrile, 20% isopropanol, 10 mmol/L ammonium formate. Mobile phase B: 90% isopropanol, 9% acetonitrile, 1% water, 10 mmol/L ammonium formate. Start (0.4 ml/min): 90% A, 0-2.7minutes: decrease to 55% A, 2.7-2.8 minutes: decrease to 47% A, 2.8-9 minutes: decrease to 35% A, 9-9.1 minutes: decrease to 11% A, 9.1-11 minutes: decrease to 8% A, 11-11.1 minutes: decrease to 0% A, 11.1 – 11.9 minutes: 0% A, 11.9 – 12 minutes: Increase to 90% A, 12 – 15 minutes: 90% A. Column temperature: 60°C. Gas temperature: 150 °C. Gas flow: 17 L/min. Nebulizer: 20 psi. Sheath gas temperature: 200 °C. Sheath gas flow: 10 L/min. Positive capillary voltage: 3500 V, Negative capillary voltage: 3000 V. Positive nozzle voltage: 1000 V. Negative nozzle voltage: 1500 V. Positive high pressure RF (iFunnel): 100 V. Negative high pressure RF (iFunnel): 90 V. Positive low pressure RF (iFunnel): 100 V. Negative low pressure RF (iFunnel): 60 V. Fragmentor: 380. Polarity: positive.

**Additional file 4C**

Amount of lipid in sample= Peak area lipid/ Peak area internal standard*amount of internal standard in sample

Proportion of lysate used to prepare sample = 40 µl / total lysate (1200 µl) = 30

Amount of lipid in lysate = Amount of lipid in sample*30

Amount of lipid per mg dry placenta = Amount of lipid in lysate/ mass dry placenta used to prepare lysate

**Additional file 4D**

Data was analyzed in R using the tidyverse, tidymodels dplyr, purrr, broom, readr, moderndive, rstatix, dlookr packages. Graphs were made using the ggplot2, ggpubr, ggrepel, ggforce, ggthemes and viridis packages.

**Additional file 4E dMRM transition list and internal standard details**

| **Lipid** | **Transition** | **Retention time (minutes)** | **Internal standard** | **Collision Energy** |
| --- | --- | --- | --- | --- |
| LPC 13:0, Avanti Polar lipids, 107 pmol per sample | 454.3 -> 184.1 | 1.64 | IS | 21 |
| LPC 22:6 | 568.3 -> 184.1 | 2.17 | LPC (13:0) | 21 |
| LPC 22:6 13C-DHA | 590.3 -> 184.1 | 2.27 | LPC (13:0) | 21 |
| LPE 14:0, Avanti Polar lipids, 114.1 pmol per sample | 426.3 -> 285.2 | 2.07 | IS | 17 |
| LPE 22:6 | 526.3 -> 385.3 | 2.28 | LPE 14:0 | 17 |
| LPE 22:6 13C-DHA | 548.3 -> 407.3 | 2.26 | LPE 14:0 | 17 |
| PC 26:0 (13:0/13:0), Avanti Polar lipids, 72.1 pmol per sample | 650.5 -> 184.1 | 4.73 | IS | 21 |
| PC 38:6 | 806.6 -> 184.1 | 6.54 | PC 26:0 | 21 |
| PC 38:6 13C-DHA | 828.6 -> 184.1 | 6.56 | PC 26:0 | 21 |
| PE(P-18:0/22:6) 13C-DHA | 798.6 -> 407.4 | 8.47 | PE 34:0 | 17 |
| PE(P-18:0/22:6) | 776.6 -> 385.4 | 8.48 | PE 34:0 | 17 |
| PE(P-16:0/22:6) | 748.5 -> 385.3 | 7.29 | PE 34:0 | 17 |
| PE(P-16:0/22:6) 13C-DHA | 770.5 -> 407.3 | 7.28 | PE 34:0 | 17 |
| DG 40:8 13C-DHA | 704.6 -> 337.3 | 8.57 | TG 51:0 | 21 |
| DG 40:8 | 682.6 -> 337.3 | 8.57 | TG 51:0 | 21 |
| PE 34:0 (17:0/17:0), Avanti Polar lipids. 100.7 pmol per sample | 720.6 -> 579.5 | 8.82 | IS | 21 |
| DG 38:6 13C-DHA | 680.5 -> 313.2 | 9.29 | TG 51:0 | 21 |
| DG 38:6 | 658.5 -> 313.2 | 9.32 | TG 51:0 | 21 |
| DG 40:7 13C-DHA | 706.6 -> 339.3 | 9.54 | TG 51:0 | 21 |
| DG 40:7 | 684.6 -> 339.3 | 9.56 | TG 51:0 | 21 |
| TG 56:9 13C-DHA | 940.8 -> 573.5 | 10.92 | TG 51:0 | 21 |
| TG 56:9 | 918.8 -> 573.5 | 10.92 | TG 51:0 | 21 |
| TG 58:10 13C-DHA | 966.8 -> 599.5 | 11.01 | TG 51:0 | 21 |
| TG 58:10 | 944.8 -> 599.5 | 11.02 | TG 51:0 | 21 |
| TG 54:7 13C-DHA | 916.8 -> 549.5 | 11.02 | TG 51:0 | 21 |
| TG 54:7 | 894.8 -> 549.5 | 11.02 | TG 51:0 | 21 |
| TG 56:8 13C-DHA | 942.8 -> 575.5 | 11.06 | TG 51:0 | 21 |
| TG 56:8 | 920.8 -> 575.5 | 11.06 | TG 51:0 | 21 |
| TG 58:9 13C-DHA | 968.8 -> 601.5 | 11.09 | TG 51:0 | 21 |
| TG 58:9 | 946.8 -> 601.5 | 11.10 | TG 51:0 | 21 |
| TG 54:6 13C-DHA | 918.8 -> 551.5 | 11.20 | TG 51:0 | 21 |
| TG 54:6 | 896.8 -> 551.5 | 11.20 | TG 51:0 | 21 |
| TG 56:7 | 922.8 -> 577.5 | 11.21 | TG 51:0 | 21 |
| TG 56:7 13C-DHA | 944.8 -> 577.5 | 11.22 | TG 51:0 | 21 |
| TG 58:8 13C-DHA | 970.9 -> 603.7 | 11.24 | TG 51:0 | 21 |
| TG 58:8 | 948.9 -> 603.7 | 11.24 | TG 51:0 | 21 |
| TG 56:6 13C-DHA | 946.8 -> 579.5 | 11.41 | TG 51:0 | 21 |
| TG 56:6 | 924.8 -> 579.5 | 11.41 | TG 51:0 | 21 |
| TG 51:0 Sigma Aldrich, 85.1 pmol per sample | 866.8 -> 579.5 | 11.84 | IS | 21 |
