# Supplementary material for: Placental 13C-DHA metabolism and relationship with maternal BMI, glycemia and birthweight
Source: Mol Med. 2021 Aug 6;27:84. doi: 10.1186/s10020-021-00344-w (PMC8349043; doi:10.1186/s10020-021-00344-w)
Supplement: Supplementary file 5 — Additional file 5. Lipid data. [file 10020_2021_344_MOESM5_ESM.docx]

**Additional file 5. Lipid data**

| **Additional file 5A. Amount in controls pmol/ dry mg** | | | | | | | | | | | | | | | | | | | | |
| --- | --- | --- | --- | --- | --- | --- | --- | --- | --- | --- | --- | --- | --- | --- | --- | --- | --- | --- | --- | --- |
| **Placenta** | **PG03** | **PG04** | **PG05** | **PG06** | **PG07** | **PG08** | **PG09** | **PG10** | **PG11** | **PG12** | **PG13** | **PG14** | **PG15** | **PG16** | **PG17** | **PG18** | **PG19** | **PG20** | **PG21** | **PG22** |
| DG 38:6 | 4.8371 | 5.7017 | 2.6338 | 4.8224 | 3.5373 | 3.0657 | 3.4719 | 3.8355 | 2.5494 | 6.6854 | 6.1740 | 1.8624 | 3.7061 | 5.7968 | 3.8297 | 7.3438 | 4.0194 | 2.5816 | 3.1322 | 4.7588 |
| DG 38:6 13C-DHA | 0.1197 | 0.1305 | 0.1069 | 0.1473 | 0.0927 | 0.0740 | 0.0932 | 0.0868 | 0.0623 | 0.1456 | 0.2291 | 0.0490 | 0.1471 | 0.1287 | 0.0858 | 0.1501 | 0.1707 | 0.0509 | 0.0528 | 0.0903 |
| DG 40:7 | 2.3722 | 2.7008 | 1.0185 | 2.0235 | 1.3721 | 1.2748 | 1.0534 | 1.4115 | 0.7492 | 2.4357 | 1.9605 | 0.6237 | 1.0770 | 2.0127 | 1.1053 | 3.0334 | 1.1430 | 0.8523 | 1.2136 | 1.8128 |
| DG 40:7 13C-DHA | 0.0477 | 0.0413 | 0.0292 | 0.0450 | 0.0260 | 0.0190 | 0.0227 | 0.0203 | 0.0132 | 0.0374 | 0.0569 | 0.0114 | 0.0394 | 0.0314 | 0.0202 | 0.0340 | 0.0417 | 0.0129 | 0.0169 | 0.0243 |
| DG 40:8 | 3.1079 | 3.1037 | 1.2188 | 2.6417 | 1.7119 | 1.5507 | 0.9671 | 1.3342 | 0.5185 | 2.1496 | 2.0566 | 0.4763 | 0.8653 | 1.9024 | 0.7867 | 2.6064 | 1.0190 | 0.6960 | 1.1988 | 1.6676 |
| DG 40:8 13C-DHA | 0.0822 | 0.0711 | 0.0556 | 0.0743 | 0.0492 | 0.0329 | 0.0298 | 0.0346 | 0.0131 | 0.0485 | 0.0790 | 0.0165 | 0.0381 | 0.0435 | 0.0201 | 0.0421 | 0.0487 | 0.0196 | 0.0227 | 0.0309 |
| LPC 22:6 | 1.7777 | 2.1947 | 1.1176 | 8.1055 | 2.9473 | 1.5136 | 4.1957 | 2.6480 | 5.6569 | 1.8274 | 2.3300 | 4.1195 | 5.9031 | 1.6119 | 4.0345 | 2.1972 | 4.7433 | 1.4519 | 0.9327 | 0.9723 |
| LPC 22:6 13C-DHA | 0.0123 | 0.0121 | 0.0129 | 0.0595 | 0.0122 | 0.0067 | 0.0251 | 0.0136 | 0.0253 | 0.0104 | 0.0242 | 0.0191 | 0.0443 | 0.0115 | 0.0176 | 0.0104 | 0.0438 | 0.0074 | 0.0046 | 0.0061 |
| LPE 22:6 | 4.8097 | 11.4686 | 6.2751 | 64.7031 | 28.7492 | 10.0697 | 37.8779 | 21.5853 | 50.3887 | 13.1648 | 12.2059 | 38.4100 | 52.8333 | 7.9567 | 35.6948 | 12.5327 | 46.8938 | 13.4754 | 4.4516 | 4.0807 |
| LPE 22:6 13C-DHA | 0.0465 | 0.0856 | 0.0542 | 0.4838 | 0.1695 | 0.0667 | 0.4329 | 0.2078 | 0.4785 | 0.1643 | 0.1657 | 0.3564 | 0.7240 | 0.0985 | 0.3863 | 0.2203 | 0.6457 | 0.1206 | 0.0436 | 0.0642 |
| PC 38:6 | 447.2412 | 350.4507 | 310.9850 | 635.9547 | 341.4022 | 308.3318 | 136.8623 | 149.2104 | 127.6828 | 173.8605 | 210.3126 | 126.0770 | 127.9276 | 217.0193 | 149.5313 | 397.2635 | 153.2704 | 166.4637 | 186.9698 | 158.2104 |
| PC 38:6 13C-DHA | 1.1750 | 0.7864 | 1.7108 | 3.3170 | 1.1020 | 0.9640 | 1.2556 | 0.7406 | 1.5324 | 0.7896 | 0.9019 | 1.1684 | 2.1848 | 0.8529 | 1.0845 | 1.1468 | 1.6795 | 0.7314 | 0.6114 | 0.6513 |
| PE-P 38:6 | 214.3636 | 211.4651 | 209.6695 | 347.6140 | 222.6303 | 187.3272 | 228.0449 | 229.7879 | 202.3733 | 343.8913 | 279.2381 | 169.2324 | 192.4973 | 249.9643 | 229.8543 | 349.8807 | 206.3104 | 260.7252 | 279.0870 | 217.6969 |
| PE-P 38:6 13C-DHA | 0.3887 | 0.3456 | 0.7285 | 1.0701 | 0.3896 | 0.3156 | 0.7237 | 0.4302 | 0.4503 | 0.8383 | 0.9622 | 0.3709 | 0.9669 | 0.5495 | 0.6445 | 0.6299 | 1.1071 | 0.3825 | 0.4640 | 0.4785 |
| PE-P 40:6 | 114.8288 | 101.2441 | 93.1260 | 157.5186 | 89.9408 | 72.6721 | 74.7524 | 81.9694 | 78.5995 | 140.7466 | 124.1740 | 68.1430 | 74.0691 | 128.3365 | 90.7256 | 122.0395 | 81.6598 | 99.7130 | 94.4326 | 96.6703 |
| PE-P 40:6 13C-DHA | 0.1509 | 0.1276 | 0.2381 | 0.3614 | 0.1226 | 0.0945 | 0.1923 | 0.1284 | 0.1334 | 0.2484 | 0.2933 | 0.1057 | 0.2767 | 0.1852 | 0.1625 | 0.1682 | 0.3145 | 0.0779 | 0.1020 | 0.1567 |
| TG 54:6 | 4.4616 | 5.0023 | 6.4206 | 14.8845 | 7.0223 | 6.4803 | 5.3396 | 6.9425 | 3.4158 | 11.0484 | 6.2825 | 3.6014 | 3.7735 | 10.8615 | 5.8169 | 11.0493 | 5.7156 | 3.1419 | 9.0525 | 10.3749 |
| TG 54:6 13C-DHA | 0.1613 | 0.1628 | 0.3264 | 0.4627 | 0.2184 | 0.1726 | 0.1776 | 0.1767 | 0.0911 | 0.3245 | 0.3030 | 0.1105 | 0.1571 | 0.2765 | 0.1523 | 0.2929 | 0.3324 | 0.0907 | 0.2433 | 0.2820 |
| TG 54:7 | 1.0462 | 1.0570 | 1.3159 | 2.2103 | 1.2894 | 1.0788 | 0.8725 | 0.9300 | 0.4823 | 2.4359 | 1.2038 | 0.5077 | 0.5953 | 1.8837 | 0.9860 | 1.6045 | 1.1130 | 0.3970 | 1.4764 | 1.3443 |
| TG 54:7 13C-DHA | 0.0399 | 0.0314 | 0.0585 | 0.0939 | 0.0409 | 0.0317 | 0.0324 | 0.0273 | 0.0184 | 0.0818 | 0.0495 | 0.0159 | 0.0192 | 0.0489 | 0.0363 | 0.0507 | 0.0641 | 0.0098 | 0.0315 | 0.0304 |
| TG 56:6 | 3.5169 | 4.7773 | 4.7711 | 9.9346 | 5.4584 | 5.2615 | 3.5758 | 4.1237 | 2.2648 | 6.5593 | 5.1183 | 2.1242 | 2.3065 | 6.9968 | 3.5883 | 6.2199 | 3.6860 | 2.2275 | 5.8166 | 6.2655 |
| TG 56:6 13C-DHA | 0.1290 | 0.1356 | 0.2409 | 0.3971 | 0.1860 | 0.1547 | 0.1063 | 0.0981 | 0.0688 | 0.2175 | 0.1767 | 0.0643 | 0.1042 | 0.1828 | 0.1068 | 0.1675 | 0.2157 | 0.0425 | 0.1405 | 0.1406 |
| TG 56:7 | 5.9980 | 6.3600 | 6.8705 | 16.1027 | 9.5330 | 7.6439 | 6.5039 | 8.1371 | 3.5755 | 15.5907 | 8.0196 | 4.3097 | 4.0348 | 11.4992 | 5.9694 | 12.9512 | 7.6524 | 3.2301 | 9.3016 | 11.0805 |
| TG 56:7 13C-DHA | 0.2104 | 0.2097 | 0.3361 | 0.5770 | 0.2915 | 0.2213 | 0.2165 | 0.2070 | 0.1238 | 0.4517 | 0.3399 | 0.1279 | 0.1405 | 0.3331 | 0.2011 | 0.3711 | 0.4073 | 0.0670 | 0.2229 | 0.3244 |
| TG 56:8 | 6.8403 | 7.0352 | 6.4124 | 16.2569 | 8.6440 | 7.6889 | 5.6285 | 7.5214 | 2.6026 | 14.9649 | 6.9446 | 3.3470 | 3.1779 | 9.5428 | 4.3722 | 11.9482 | 7.0659 | 3.0835 | 9.2959 | 8.4784 |
| TG 56:8 13C-DHA | 0.2544 | 0.2375 | 0.3121 | 0.7520 | 0.3145 | 0.2581 | 0.1910 | 0.2297 | 0.0969 | 0.4394 | 0.3457 | 0.0993 | 0.1758 | 0.3352 | 0.1349 | 0.3307 | 0.4270 | 0.0876 | 0.2355 | 0.2787 |
| TG 56:9 | 0.9760 | 0.8893 | 0.8188 | 1.4098 | 1.0690 | 0.6811 | 0.4660 | 0.5744 | 0.1936 | 1.2571 | 0.6526 | 0.2213 | 0.2683 | 0.9244 | 0.4076 | 0.9379 | 0.5670 | 0.2473 | 0.8112 | 0.8399 |
| TG 56:9 13C-DHA | 0.0413 | 0.0336 | 0.0458 | 0.0631 | 0.0380 | 0.0210 | 0.0166 | 0.0175 | 0.0054 | 0.0312 | 0.0426 | 0.0080 | 0.0152 | 0.0306 | 0.0152 | 0.0292 | 0.0326 | 0.0058 | 0.0287 | 0.0368 |
| TG 58:8 | 2.8606 | 3.0972 | 3.1607 | 7.2367 | 3.7239 | 4.0706 | 2.4684 | 3.1549 | 1.2857 | 6.7485 | 3.3844 | 1.4837 | 1.5554 | 4.3312 | 2.1031 | 6.1717 | 2.8670 | 1.7978 | 3.3482 | 3.8517 |
| TG 13C-DHA | 0.0725 | 0.0681 | 0.1381 | 0.2235 | 0.0981 | 0.0998 | 0.0703 | 0.0711 | 0.0439 | 0.1439 | 0.1197 | 0.0285 | 0.0601 | 0.0974 | 0.0462 | 0.0961 | 0.1399 | 0.0372 | 0.0665 | 0.0734 |
| TG 58:9 | 5.6027 | 5.0978 | 5.2599 | 11.1843 | 6.4928 | 6.2267 | 3.7761 | 4.9148 | 1.9347 | 10.1116 | 5.7083 | 2.2556 | 2.4436 | 7.7015 | 3.2689 | 8.1380 | 3.9222 | 2.5404 | 5.4303 | 7.0157 |
| TG 58:9 13C-DHA | 0.1775 | 0.1397 | 0.2520 | 0.4082 | 0.1260 | 0.1574 | 0.1420 | 0.1155 | 0.0582 | 0.2452 | 0.2355 | 0.0550 | 0.1097 | 0.2092 | 0.1080 | 0.2063 | 0.2258 | 0.0715 | 0.1352 | 0.1864 |
| TG 58:10 | 7.8622 | 7.6816 | 7.5766 | 13.5943 | 9.8028 | 7.1656 | 4.6541 | 5.6454 | 1.7299 | 10.7522 | 6.5923 | 2.7055 | 2.7103 | 8.1891 | 3.9939 | 9.7002 | 5.2054 | 4.0620 | 8.2529 | 11.8990 |
| TG 58:10 13C-DHA | 0.2876 | 0.2320 | 0.3962 | 0.5461 | 0.2974 | 0.2299 | 0.1684 | 0.1525 | 0.0623 | 0.3206 | 0.3122 | 0.0872 | 0.1232 | 0.3097 | 0.1478 | 0.3002 | 0.2970 | 0.1268 | 0.2031 | 0.3230 |

| **Additional file 5B. Amount in glucose treated explants pmol/ dry mg** | | | | | | | | | | | | | | | | | | | | |
| --- | --- | --- | --- | --- | --- | --- | --- | --- | --- | --- | --- | --- | --- | --- | --- | --- | --- | --- | --- | --- |
| **Placenta** | **PG03** | **PG04** | **PG05** | **PG06** | **PG07** | **PG08** | **PG09** | **PG10** | **PG11** | **PG12** | **PG13** | **PG14** | **PG15** | **PG16** | **PG17** | **PG18** | **PG19** | **PG20** | **PG21** | **PG22** |
| DG 38:6 | 3.6966 | 4.3576 | 2.4610 | 4.2769 | 3.4827 | 2.6943 | 3.3953 | 4.8261 | 2.8984 | 2.7261 | 7.6705 | 1.6926 | 3.1635 | 3.6654 | 3.8795 | 6.5684 | 4.4004 | 3.0469 | 3.7771 | 3.1970 |
| DG 38:6 13C-DHA | 0.1023 | 0.1434 | 0.0973 | 0.1073 | 0.1103 | 0.0528 | 0.1537 | 0.1613 | 0.0486 | 0.0553 | 0.3137 | 0.0476 | 0.1152 | 0.0935 | 0.0715 | 0.1288 | 0.1635 | 0.0428 | 0.0924 | 0.0712 |
| DG 40:7 | 1.8074 | 1.7193 | 0.9328 | 1.8005 | 1.2674 | 1.2134 | 1.0139 | 1.6810 | 0.9545 | 0.9350 | 2.3419 | 0.5466 | 1.0225 | 1.2693 | 1.0449 | 2.7106 | 1.1575 | 0.9850 | 1.4094 | 1.0681 |
| DG 40:7 13C-DHA | 0.0368 | 0.0459 | 0.0313 | 0.0302 | 0.0317 | 0.0172 | 0.0366 | 0.0446 | 0.0135 | 0.0138 | 0.0902 | 0.0121 | 0.0317 | 0.0223 | 0.0150 | 0.0364 | 0.0327 | 0.0113 | 0.0284 | 0.0152 |
| DG 40:8 | 2.4512 | 2.0938 | 1.1243 | 2.5680 | 1.4194 | 1.4549 | 1.0238 | 1.5754 | 0.7172 | 0.7511 | 2.5216 | 0.4662 | 0.7819 | 1.0422 | 0.8385 | 2.3815 | 1.0316 | 0.8766 | 1.3560 | 0.9574 |
| DG 40:8 13C-DHA | 0.0630 | 0.0743 | 0.0566 | 0.0618 | 0.0467 | 0.0320 | 0.0478 | 0.0623 | 0.0152 | 0.0156 | 0.1105 | 0.0136 | 0.0287 | 0.0314 | 0.0181 | 0.0488 | 0.0436 | 0.0177 | 0.0371 | 0.0241 |
| LPC 22:6 | 1.2618 | 5.2611 | 1.7680 | 3.1647 | 2.8752 | 1.0484 | 3.4058 | 4.7674 | 2.1894 | 2.1346 | 2.6346 | 2.8100 | 2.8926 | 0.8392 | 3.1128 | 1.4768 | 5.8894 | 2.0139 | 0.9433 | 0.9640 |
| LPC 22:6 13C-DHA | 0.0117 | 0.0316 | 0.0154 | 0.0276 | 0.0183 | 0.0060 | 0.0316 | 0.0292 | 0.0120 | 0.0084 | 0.0361 | 0.0177 | 0.0217 | 0.0070 | 0.0120 | 0.0072 | 0.0406 | 0.0059 | 0.0077 | 0.0065 |
| LPE 22:6 | 3.7307 | 42.1454 | 11.3597 | 21.1199 | 26.1538 | 5.5371 | 40.8552 | 45.6872 | 19.0365 | 20.4638 | 15.0831 | 26.5911 | 26.1156 | 4.1819 | 25.9407 | 7.3020 | 55.3460 | 26.1493 | 4.3223 | 4.2284 |
| LPE 22:6 13C-DHA | 0.0314 | 0.3834 | 0.1122 | 0.1363 | 0.1915 | 0.0467 | 0.5503 | 0.5385 | 0.1641 | 0.2141 | 0.2405 | 0.2648 | 0.2930 | 0.0760 | 0.2725 | 0.1231 | 0.6375 | 0.1969 | 0.0608 | 0.0572 |
| PC 38:6 | 355.8095 | 389.1289 | 282.6640 | 450.3958 | 287.3460 | 274.0889 | 91.5611 | 179.5411 | 116.8396 | 102.5649 | 217.7659 | 99.5052 | 82.6300 | 120.3649 | 168.4526 | 383.7404 | 146.6579 | 222.2529 | 176.4759 | 127.1122 |
| PC 38:6 13C-DHA | 0.9215 | 1.6761 | 1.5489 | 1.7658 | 1.1729 | 0.7895 | 1.3703 | 1.5338 | 0.5244 | 0.5424 | 1.5992 | 0.7545 | 0.8469 | 0.4832 | 0.8528 | 1.3960 | 1.6408 | 0.8315 | 0.6167 | 0.5175 |
| PE-P 38:6 | 197.8212 | 204.7307 | 216.2735 | 302.1283 | 184.3038 | 168.2455 | 157.3056 | 274.7957 | 222.0766 | 170.4504 | 299.2944 | 155.1134 | 136.4416 | 166.9436 | 234.7493 | 340.0000 | 175.7405 | 253.1890 | 306.8403 | 177.7405 |
| PE-P 38:6 13C-DHA | 0.3279 | 0.6620 | 0.6189 | 0.6227 | 0.5158 | 0.2768 | 0.9657 | 1.0519 | 0.3176 | 0.3453 | 1.2538 | 0.3332 | 0.5494 | 0.3925 | 0.4459 | 0.6874 | 0.5985 | 0.2701 | 0.8169 | 0.3597 |
| PE-P 40:6 | 91.3721 | 83.3037 | 92.2165 | 140.2068 | 78.4766 | 71.6806 | 51.7455 | 98.2089 | 88.8456 | 68.5526 | 147.3729 | 62.4163 | 52.8869 | 82.6184 | 89.6200 | 128.1164 | 72.8348 | 98.2454 | 104.0341 | 79.9099 |
| PE-P 40:6 13C-DHA | 0.1222 | 0.2275 | 0.2150 | 0.2242 | 0.1501 | 0.0825 | 0.2390 | 0.3055 | 0.1028 | 0.0733 | 0.4560 | 0.0994 | 0.1821 | 0.1378 | 0.1107 | 0.1615 | 0.1799 | 0.0550 | 0.2048 | 0.1103 |
| TG 54:6 | 4.4982 | 6.1012 | 6.4146 | 10.9089 | 8.9269 | 7.8504 | 4.2361 | 8.0956 | 5.0204 | 4.1075 | 8.4371 | 3.1549 | 2.9807 | 7.9028 | 6.4590 | 11.0124 | 4.1311 | 3.2215 | 11.5881 | 6.6655 |
| TG 54:6 13C-DHA | 0.1393 | 0.2599 | 0.2688 | 0.3040 | 0.2955 | 0.1565 | 0.1911 | 0.3037 | 0.1216 | 0.1081 | 0.3426 | 0.1080 | 0.0963 | 0.2035 | 0.1559 | 0.2566 | 0.1570 | 0.0608 | 0.3856 | 0.2064 |
| TG 54:7 | 1.1286 | 1.4037 | 1.2584 | 1.8920 | 1.3438 | 1.2150 | 0.7587 | 1.2279 | 0.7620 | 0.7582 | 1.3703 | 0.5158 | 0.3820 | 1.2211 | 0.9920 | 1.7387 | 0.5878 | 0.4505 | 1.7894 | 0.9002 |
| TG 54:7 13C-DHA | 0.0331 | 0.0620 | 0.0494 | 0.0586 | 0.0612 | 0.0276 | 0.0440 | 0.0533 | 0.0231 | 0.0240 | 0.0720 | 0.0180 | 0.0130 | 0.0386 | 0.0189 | 0.0473 | 0.0259 | 0.0040 | 0.0595 | 0.0257 |
| TG 56:6 | 3.7720 | 4.4124 | 4.9383 | 8.2762 | 5.7444 | 5.3074 | 3.0468 | 5.3495 | 2.9149 | 2.3968 | 6.2059 | 1.9187 | 1.8450 | 4.3793 | 4.2949 | 5.8323 | 2.5377 | 2.2149 | 8.7668 | 3.6313 |
| TG 56:6 13C-DHA | 0.1309 | 0.1923 | 0.2254 | 0.2661 | 0.1881 | 0.1247 | 0.1302 | 0.1988 | 0.0744 | 0.0576 | 0.2507 | 0.0586 | 0.0723 | 0.1214 | 0.0984 | 0.1520 | 0.1001 | 0.0461 | 0.2150 | 0.1049 |
| TG 56:7 | 5.9524 | 7.8483 | 6.8431 | 12.1005 | 10.8897 | 8.7855 | 5.1210 | 9.6871 | 6.0420 | 4.9486 | 9.1148 | 4.0729 | 3.1608 | 8.4691 | 6.6618 | 13.3964 | 5.0114 | 3.4185 | 13.0573 | 7.6865 |
| TG 56:7 13C-DHA | 0.2036 | 0.3667 | 0.2795 | 0.3858 | 0.3587 | 0.2161 | 0.2525 | 0.3948 | 0.1647 | 0.1212 | 0.4042 | 0.1365 | 0.1213 | 0.2331 | 0.1664 | 0.3674 | 0.2176 | 0.0689 | 0.4170 | 0.2141 |
| TG 56:8 | 7.0904 | 8.1999 | 6.2236 | 13.6943 | 10.5625 | 7.7401 | 4.3703 | 9.7244 | 4.7960 | 4.8734 | 7.9193 | 2.9059 | 2.5334 | 7.1228 | 5.4250 | 13.4239 | 4.6220 | 3.1333 | 11.7605 | 5.8596 |
| TG 56:8 13C-DHA | 0.2383 | 0.3503 | 0.3243 | 0.5359 | 0.3443 | 0.2530 | 0.2601 | 0.4014 | 0.1299 | 0.1203 | 0.4207 | 0.1071 | 0.1072 | 0.2439 | 0.1111 | 0.4138 | 0.2139 | 0.0716 | 0.3908 | 0.1836 |
| TG 56:9 | 0.9082 | 0.8042 | 0.7697 | 1.2571 | 0.8867 | 0.6525 | 0.4313 | 0.7693 | 0.3374 | 0.4008 | 0.7598 | 0.2268 | 0.2544 | 0.5463 | 0.4118 | 0.9594 | 0.3357 | 0.2212 | 1.0587 | 0.4824 |
| TG 56:9 13C-DHA | 0.0302 | 0.0415 | 0.0428 | 0.0494 | 0.0350 | 0.0224 | 0.0267 | 0.0348 | 0.0100 | 0.0082 | 0.0458 | 0.0091 | 0.0109 | 0.0203 | 0.0114 | 0.0284 | 0.0128 | 0.0050 | 0.0469 | 0.0167 |
| TG 58:8 | 3.1317 | 3.5694 | 2.8274 | 5.8515 | 4.7503 | 3.9172 | 2.3857 | 4.2967 | 2.0582 | 2.0615 | 3.8133 | 1.3555 | 1.0986 | 2.7615 | 2.0959 | 6.6958 | 1.6521 | 1.7744 | 5.3703 | 2.3081 |
| TG 13C-DHA | 0.0791 | 0.0934 | 0.0851 | 0.0897 | 0.1384 | 0.0875 | 0.0924 | 0.1203 | 0.0553 | 0.0433 | 0.1693 | 0.0293 | 0.0343 | 0.0812 | 0.0494 | 0.1235 | 0.0598 | 0.0329 | 0.1225 | 0.0565 |
| TG 58:9 | 5.5378 | 5.6078 | 4.7173 | 8.4987 | 7.3330 | 6.8942 | 3.1921 | 6.1346 | 3.5378 | 3.0330 | 6.4089 | 1.9792 | 1.7825 | 5.1005 | 3.4609 | 9.6010 | 2.5586 | 2.5628 | 7.5646 | 4.5685 |
| TG 58:9 13C-DHA | 0.1604 | 0.2309 | 0.2504 | 0.2672 | 0.2521 | 0.1574 | 0.1447 | 0.2317 | 0.0993 | 0.0756 | 0.3378 | 0.0742 | 0.0724 | 0.1718 | 0.0893 | 0.2227 | 0.0738 | 0.0650 | 0.2423 | 0.1281 |
| TG 58:10 | 7.7265 | 7.2905 | 7.2363 | 13.4708 | 8.7369 | 7.1875 | 3.7533 | 6.6935 | 3.3693 | 3.3101 | 7.4888 | 2.5763 | 1.9184 | 5.5743 | 4.6522 | 11.1776 | 3.0424 | 3.7620 | 11.1605 | 6.6151 |
| TG 58:10 13C-DHA | 0.2856 | 0.3683 | 0.3575 | 0.4724 | 0.3371 | 0.2119 | 0.2028 | 0.2861 | 0.1003 | 0.0861 | 0.3964 | 0.0906 | 0.0992 | 0.1949 | 0.1280 | 0.3144 | 0.1314 | 0.0928 | 0.3988 | 0.2259 |
