# Supplementary material for: Placental 13C-DHA metabolism and relationship with maternal BMI, glycemia and birthweight
Source: Mol Med. 2021 Aug 6;27:84. doi: 10.1186/s10020-021-00344-w (PMC8349043; doi:10.1186/s10020-021-00344-w)
Supplement: Supplementary file 6 — Additional file 6. The association of post-load glycemia with the enrichment of placental 13C-DHA lipids (Z-score, log2 transformed) for 17 placenta. [file 10020_2021_344_MOESM6_ESM.docx]

**Additional file 6. The association of post-load glycemia with the *enrichment* of placental ^13^C-DHA lipids (Z-score, Log2 transformed) for 17 placenta**


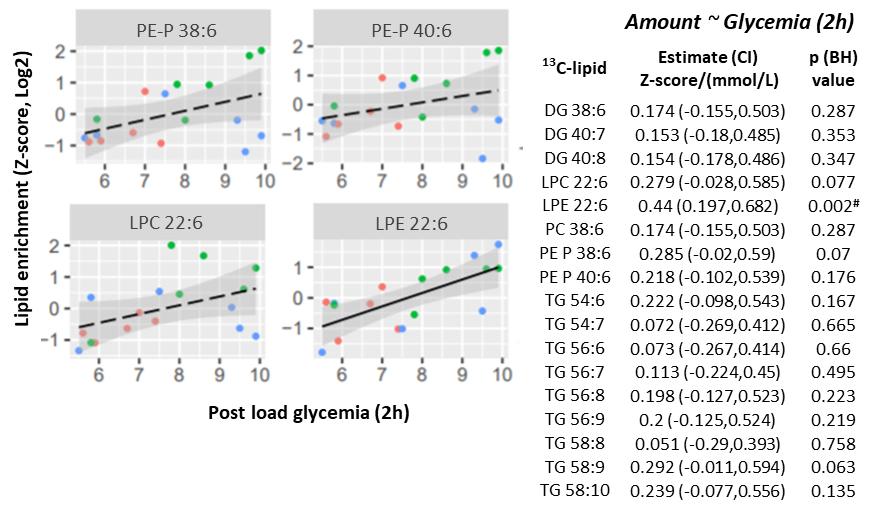


Additional file 6. The association of post-load glycemia with the *enrichment* of placental ^13^C-DHA lipids (Z-score, Log2 transformed) for 17 placenta. Linear regression was run with lipid as the outcome and clinical characteristic as the variable. The Benjamini-Hochberg method was used to correct for multiple testing. Solid lines show significant associations while dashed lines show non-significant associations. Shaded areas show 95% confidence intervals. Key - Purple: non GDM, Black: GDM. ^#^Significant after adjusting for BMI. BH: Benjamini-Hochberg, CI: Confidence interval, DG: diacylglycerol, DHA: Docosahexaenoic acid, GDM: Gestational diabetes, LPC: lyso-phosphatidylcholine, LPE: lyso-phosphatidylethanolamine, PC: phosphatidylcholine, PE-P: phosphatidylethanolamine-plasmalogen, TG: triacylglycerol.
