# Supplementary material for: Placental 13C-DHA metabolism and relationship with maternal BMI, glycemia and birthweight
Source: Mol Med. 2021 Aug 6;27:84. doi: 10.1186/s10020-021-00344-w (PMC8349043; doi:10.1186/s10020-021-00344-w)
Supplement: Supplementary file 7 — Additional file 7. Associations (linear regression) between maternal metabolic factors and lipid amount (Z-score, log2) after mutual adjustment [file 10020_2021_344_MOESM7_ESM.docx]

**Additional file 7. Associations (linear regression) between maternal metabolic factors and lipid amount (Z-score, log2) after mutual adjustment**

| **Associations (linear regression) between maternal metabolic factors and lipid amount (Z-score, log2) after mutual adjustment** | | | | |
| --- | --- | --- | --- | --- |
| **^13^C-DHA lipid** | **Association with BMI after adjustment for fasting glycemia**  **Estimate (lower CI, upper CI)**  **Z-score/(kg/m^2^)** | **p value (BH)** | **Association with fasting glycemia after adjustment for BMI**  **Estimate (lower CI, upper CI)**  **Z-score/(mmol/L)** | **p value (BH)** |
| DG 38:6 | 0.063 (-0.051,0.177) | 0.287 | 0.592 (-0.555,1.739) | 0.287 |
| DG 40:7 | 0.038 (-0.081,0.157) | 0.503 | 0.494 (-0.703,1.692) | 0.503 |
| DG 40:8 | -0.019 (-0.142,0.104) | 0.743 | 0.538 (-0.7,1.777) | 0.738 |
| LPC 22:6 | 0.109 (-0.016,0.234) | 0.252 | 0.111 (-1.147,1.37) | 0.852 |
| LPE 22:6 | 0.09 (-0.038,0.217) | 0.459 | -0.034 (-1.315,1.247) | 0.955 |
| PC 38:6 | 0.101 (-0.027,0.229) | 0.336 | -0.005 (-1.291,1.281) | 0.993 |
| PE-P 38:6 | 0.114 (0.004,0.225) | 0.100 | 0.37 (-0.743,1.483) | 0.488 |
| PE-P 40:6 | 0.113 (0.006,0.221) | 0.062 | 0.526 (-0.557,1.61) | 0.315 |
| TG 54:6 | -0.009 (-0.122,0.104) | 0.866 | 1.121 (-0.013,2.255) | 0.093 |
| TG 54:7 | -0.027 (-0.132,0.079) | 0.596 | 1.212 (0.148,2.277) | 0.071 |
| TG 56:6 | -0.015 (-0.132,0.102) | 0.783 | 1.013 (-0.163,2.19) | 0.170 |
| TG 56:7 | -0.026 (-0.136,0.085) | 0.627 | 1.246 (0.135,2.357) | 0.075 |
| TG 56:8 | -0.036 (-0.139,0.066) | 0.460 | 1.176 (0.14,2.211) | 0.087 |
| TG 56:9 | -0.04 (-0.162,0.082) | 0.495 | 0.828 (-0.403,2.058) | 0.483 |
| TG 58:8 | -0.014 (-0.115,0.086) | 0.762 | 0.975 (-0.034,1.983) | 0.123 |
| TG 58:9 | -0.027 (-0.13,0.076) | 0.584 | 1.137 (0.096,2.177) | 0.096 |
| TG 58:10 | -0.054 (-0.164,0.057) | 0.316 | 1.098 (-0.015,2.211) | 0.159 |

BH: Benjamini-Hochberg corrected
