# Supplementary material for: Placental 13C-DHA metabolism and relationship with maternal BMI, glycemia and birthweight
Source: Mol Med. 2021 Aug 6;27:84. doi: 10.1186/s10020-021-00344-w (PMC8349043; doi:10.1186/s10020-021-00344-w)
Supplement: Supplementary file 8 — Additional file 8. The association of 13C-DHA-lipid amount (Z-score, Log2) with birthweight centile for 17 placenta. [file 10020_2021_344_MOESM8_ESM.docx]

**Additional file 8. The association of ^13^C-DHA-lipid amount (Z-score, Log2) with birthweight centile for 17 placenta.**

**
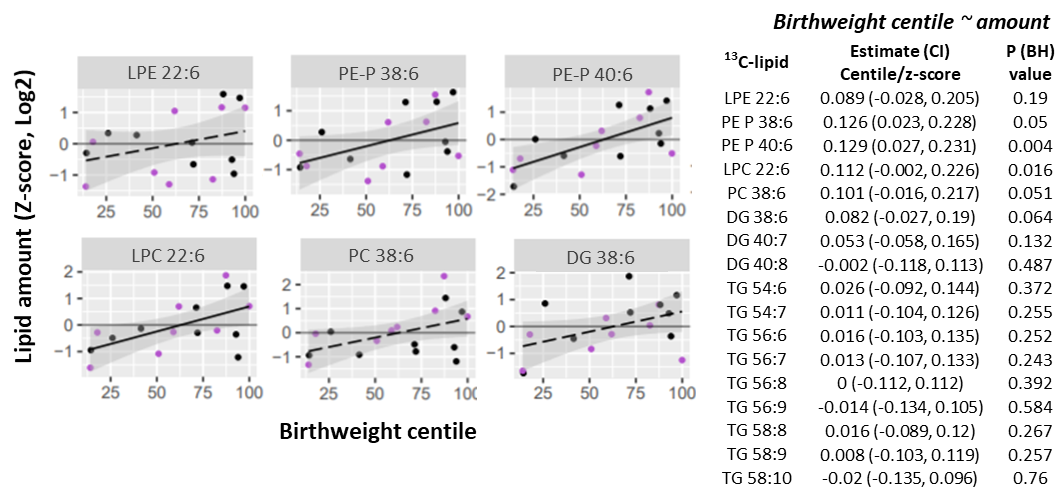
**

Additional file 8. The association of ^13^C-DHA-lipid amount (Z-score, Log2) with birthweight centile for 17 placenta. Linear regression was run with birthweight centile as the outcome and either lipid amount or lipid enrichment as the predictor. The Benjamini-Hochberg method was used to correct for multiple testing. Solid lines show significant associations while dashed lines show non-significant results. Shaded areas show 95% confidence intervals. Key - Purple: non-GDM, Black: GDM. BH: Benjamini-Hochberg CI: Confidence interval, DG: diacylglycerol, DHA: Docosahexaenoic acid, GDM: Gestational diabetes, LPC: lyso-phosphatidylcholine, LPE: lyso-phosphatidylethanolamine, PC: phosphatidylcholine, PE-P: phosphatidylethanolamine-plasmalogen, TG: triacylglycerol
