# Supplementary material for: Placental 13C-DHA metabolism and relationship with maternal BMI, glycemia and birthweight
Source: Mol Med. 2021 Aug 6;27:84. doi: 10.1186/s10020-021-00344-w (PMC8349043; doi:10.1186/s10020-021-00344-w)
Supplement: Supplementary file 9 — Additional file 9. Association between BMI (Z-score, log2) and birthweight centile adjusted for each DHA lipid. [file 10020_2021_344_MOESM9_ESM.docx]

**Additional file 9. Association between BMI (Z-score, log2) and birthweight centile adjusted for each DHA lipid**

| **Association between BMI (Z-score, log2) and birthweight centile adjusted for each DHA lipid** | | | | |
| --- | --- | --- | --- | --- |
| **Prior to lipid adjustment** | **estimate (CI) = 19.9 (7.2, 32.7), p=0.004** | | | |
| **^13^C-DHA lipid** | **Adjusted for ^13^C-DHA lipid enrichment (Z-score, log2)** | | **Adjusted for ^13^C-DHA lipid amount (Z-score, log2)** | |
|  | **estimate (lower CI, upper CI)**  **Centile/Z-score** | **p value (BH)** | **estimate (lower CI, upper CI)**  **Centile/Z-score** | **p value (BH)** |
| DG 38:6 | 16.069 (0.169,31.969) | 0.072 | 17.127 (3.436,30.818) | 0.027 |
| DG 40:7 | 16.979 (0.387,33.572) | 0.069 | 18.197 (5.093,31.3) | 0.015 |
| DG 40:8 | 19.346 (3.464,35.228) | 0.03 | 20.042 (7.213,32.87) | 0.0075 |
| LPC 22:6 | 14.625 (-0.421,29.671) | 0.084 | 15.025 (1.209,28.841) | 0.0525 |
| LPE 22:6 | 20.629 (5.525,35.732) | 0.0165 | 18.848 (4.58,33.115) | 0.0195 |
| PC 38:6 | 16.852 (-0.434,34.138) | 0.0825 | 16.767 (2.735,30.799) | 0.0345 |
| PE-P 38:6 | 11.941 (-5.175,29.056) | 0.168 | 17.072 (1.387,32.757) | 0.0525 |
| PE-P 40:6 | 12.103 (-3.013,27.218) | 0.108 | 12.65 (-1.752,27.053) | 0.081 |
| TG 54:6 | 18.725 (3.671,33.779) | 0.027 | 19.409 (6.351,32.468) | 0.0105 |
| TG 54:7 | 17.008 (3.65,30.367) | 0.024 | 19.566 (7.102,32.03) | 0.0075 |
| TG 56:6 | 16.413 (1.387,31.439) | 0.051 | 19.415 (6.856,31.974) | 0.0075 |
| TG 56:7 | 12.936 (-0.516,26.389) | 0.058 | 19.497 (7.041,31.953) | 0.0075 |
| TG 56:8 | 10.723 (-6.047,27.494) | 0.192 | 19.975 (7.311,32.638) | 0.006 |
| TG 56:9 | 15.956 (1.176,30.736) | 0.054 | 20.35 (7.486,33.215) | 0.006 |
| TG 58:8 | 14.499 (-1.359,30.357) | 0.105 | 19.386 (6.735,32.036) | 0.0075 |
| TG 58:9 | 13.798 (-1.264,28.86) | 0.105 | 19.647 (7.229,32.064) | 0.006 |
| TG 58:10 | 13.584 (-3.585,30.753) | 0.168 | 20.375 (7.306,33.444) | 0.0075 |

CI: confidence interval, BH: Benjamini-Hochberg corrected
